# Clitoris Anatomy Applied to Aesthetic, Reconstructive and Transgender Surgery: A Narrative Review

**DOI:** 10.1590/S1677-5538.IBJU.2026.9910

**Published:** 2026-06-10

**Authors:** Luciano A. Favorito

**Affiliations:** 1 Universidade do Estado do Rio de Janeiro Unidade de Pesquisa Urogenital Rio de Janeiro RJ Brasil Unidade de Pesquisa Urogenital da Universidade do Estado do Rio de Janeiro - UERJ, Rio de Janeiro, RJ, Brasil; 2 Universidade Estácio de Sá Instituto de Educação Médica Rio de Janeiro RJ Brasil Instituto de Educação Médica - IDOMED, Universidade Estácio de Sá - UNESA, Rio de Janeiro, RJ, Brasil

**Keywords:** Clitoris, Anatomy, Surgery, Plastic

## Abstract

This review aims to provide a comprehensive analysis of the anatomy of the clitoris, focusing on its complex internal structure, topographic relationships, and neurovascular supply highlighting its morphological features and clinical implications in aesthetic, reconstructive and transgender surgery. We analyzed papers published in the past 70 years in the databases of Pubmed, Embase and Scielo. We excluded case reports, editorials and opinions of specialists. Studies were excluded if they lacked clear anatomical descriptions of the clitoris, if full-text access was unavailable, or if they were published in languages other than Portuguese, English, or Spanish. Long overshadowed by other anatomical studies, the clitoris is now understood as a multi-planar organ with significant depth and extensive innervation with several applications and surgical techniques, such as clitoral hood reduction (hoodectomy) or labiaplasty. The clitoris is a very important structure to female physiology, specially orgasm. Knowledge of clitoris anatomy is a fundamental step in aesthetic surgery and in reconstruction. In aesthetic and reconstructive clitoroplasty, Buck's fascia is a critical anatomical landmark. The neurovascular bundle resides just beneath this fascia on the dorsal aspect of the clitoris. By staying superficial to or carefully dissecting within this layer, surgeons can ensure that the blood supply and sensory fibers remain intact, preventing glans necrosis and desensitization. This narrative review addresses the anatomical integration, surgical techniques, and functional importance of the clitoral complex in the transgender population and transgender surgery.

## INTRODUCTION

The vulva consists of a series of structures located in the anterior region of the perineum. It is the site where the main procedures in regenerative gynecological surgery are performed. The vulva can be didactically divided into 6 regions or complexes: Pubic area; Clitoral complex; Clitorolabial interface; Labia minora; Labia majora and Perineal region. This division is of great importance in the study of the perineal and vulvar region (1). In the field of aesthetic surgery, the primary goal is to enhance the appearance of the vulva while strictly preserving sensation and function. In this review we will describe the anatomy of clitoral complex and clitorolabial interface.

Understanding clitoral topography is vital in surgical procedures such as vulvectomies, pelvic floor reconstructions, and gender-affirming surgeries. The clitoris is not merely a superficial glans; it is a complex, tri-dimensional structure composed of the glans, the body (corpus), and the bilateral crura (2). Understanding the precise pathways of the dorsal nerves and the deep arteries is critical to avoid complications like chronic pain, numbness, or tissue necrosis and to preserving sexual function and sensation. In this review we show the most important aspects of the clitoris surgical anatomy applied to aesthetic, reconstructive and transgender surgery.

## CLITORIS EMBRIOLOGY

In embryology of the female genital tract, both the external and internal genitalia differ before the end of the first trimester. The female genitals are made up of the gonads, fallopian tubes, uterus, cervix, vagina and external genitals. A male fetus develops in the presence of a Y chromosome that encodes the SRY protein. The SRY protein allows for testicular differentiation and androgen production, including testosterone. In its absence, this process will not occur, leading to the formation of a female fetus (3, 4).

Embryological development of the female reproductive tract is a process rich in its complexity in which fetal structures are differentiated in an orchestrated way to form external genitalia (4, 5). Any impairment at this stage can lead to a slight difference, resulting in congenital anomalies that affect the female genital tract. The genital tubercle is the anlage of the external genitalia in both males and females. Congenital abnormalities frequently result from abnormal formation of this structure and its subsequent development (3-6). The most important parameter in female genitalia to evaluate the fetal androgen exposure and gender detection between 11 and 13 weeks post conception (WPC) is the AGD (anogenital distance) (6). The relationship between AGD and serum testosterone in both genders was reported, meaning that a long distance is associated with elevated serum testosterone levels (5). The female external genitalia do not require fetal ovarian hormones in their development (4-6) as occurs in male fetuses that requires hormones for their development.

In early development, the external genitalia are undifferentiated. The genital tubercle initiates differentiation into a penis or clitoris at 8 WPC. The female external genitalia develop due to hormonal stimuli between the third and fifth month of gestation (5-7). The genital tubercle elongates, giving rise to the clitoris; the urethral folds do not fuse and give rise to the labia minora. The genital folds elongate and form the labia majora; the urogenital sulcus remains open and forms the vaginal vestibule (Figure-1). In an interesting paper with human female fetuses using reconstruction and histological techniques shows important aspects of the clitoris development (7). The authors show that the mesenchymal inside the clitoris soon divides into the glans and the cavernous bodies (7). The clitoral hood forms between 10 and 14 WPC as a fold of tissue that extends from proximal to distal over the glans. The labia majora gradually cover the clitoris after 14 WPC. The labia minora form at 8 WPC from the ridges of thick-skin epithelium that flank the genital exit. In this paper the authors proposed that the clitoris "disappears" between both labia majora because the 50% of decline in its length between 15 and 20 WPC (7). However, when we analyzed our sample in a previous publication with 22 female fetuses we observed that there are no significant differences in clitoris length and width between 12 and 22 WPC and the glans clitoris is easily observed in female human fetuses during the 2nd gestational trimester (Figure-2) (8). Normative curves about the clitoris development are scarce in literature but has great importance to identify clitoromegaly in neonatal period (9).

**Figure 1:**
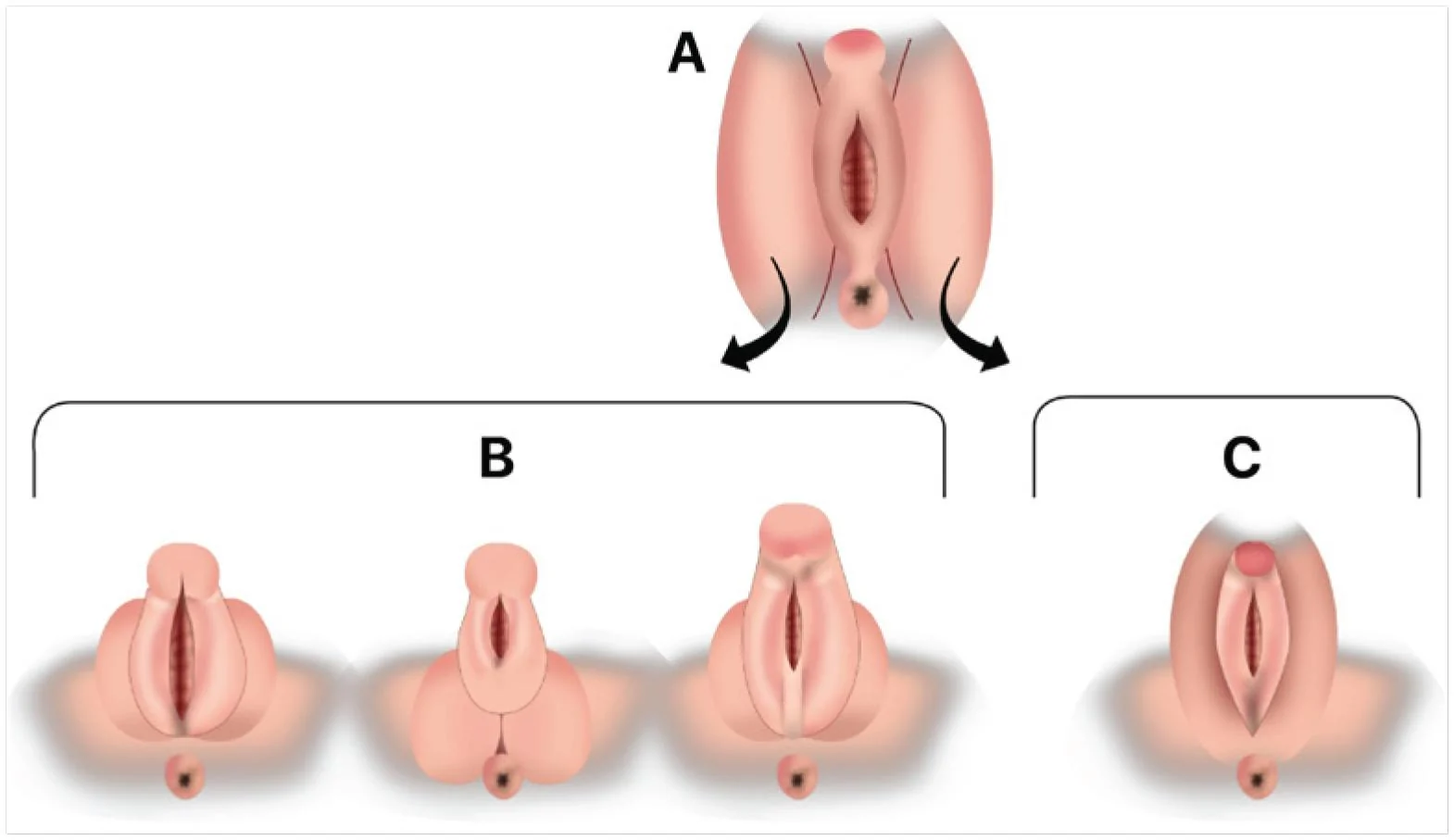
Clitoris Embryology.

Schematic drawing showing the genital development in male and in female. A) Undifferentiated stage; B) Male genitalia development, we can observe the elongation of genital tubercle, to form the penis and C) Female genitalia totally developed.

**Figure 2:**
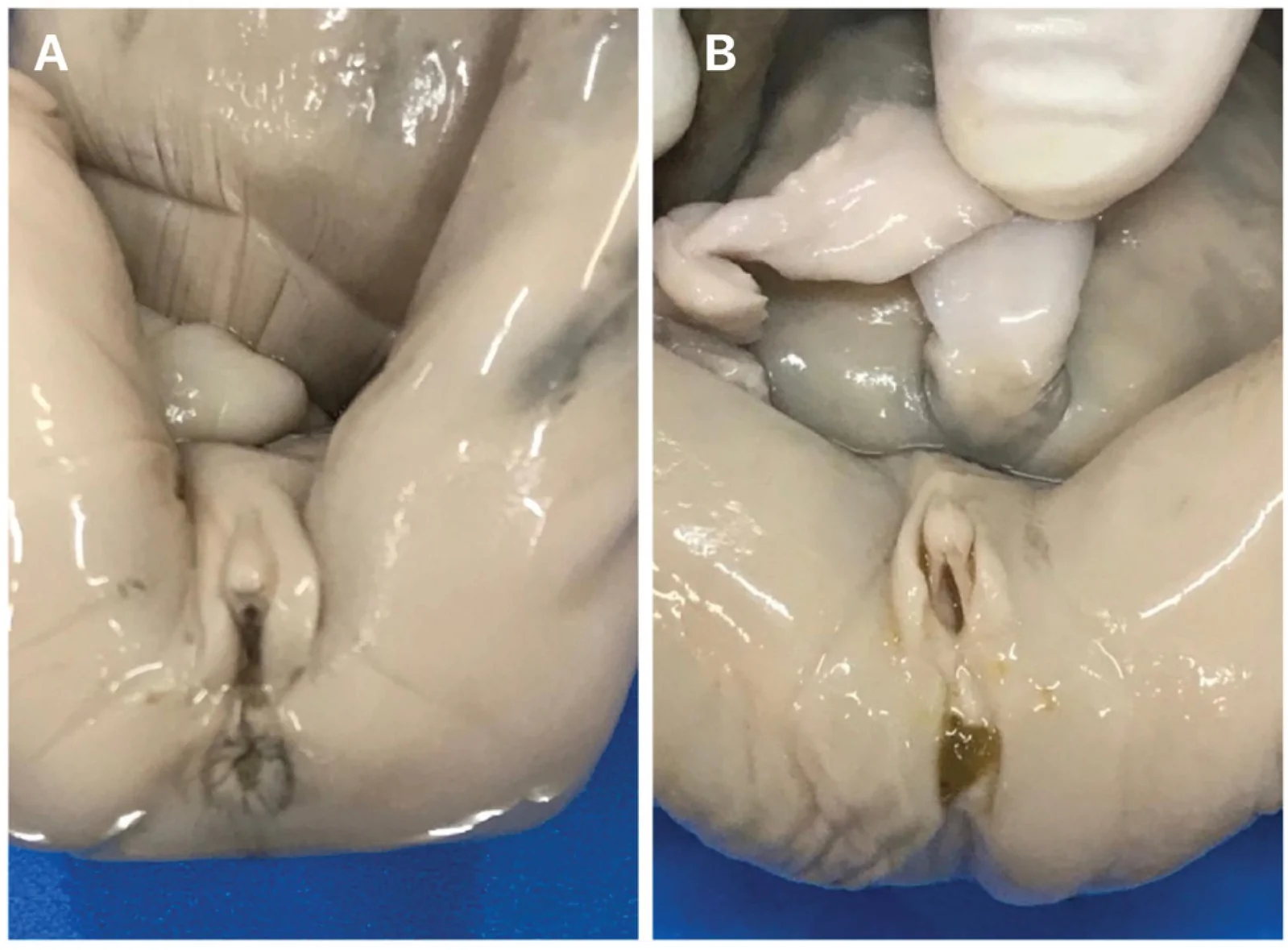
Clitoris in 2nd gestational trimester in human fetuses.

The figure shows two female fetuses with 16weeks post-conception (A) and 18weeks post-conception (B). We can observe the clitoris aspect during the 2nd gestational trimester. In this period the glans and clitoris frenulum are easily identified.

A nice basic research study of human fetal genital tubercles during the first trimester after elective termination of pregnancy showed that the distinction of glans and shaft begins at 9 WPC and the formation of the prepuce starts to cover the dorsal aspect of the glans clitoris at 11 to 12 WPC. The authors also identified the development of the clitoral ridge as a well vascularized structure at the midline of the vestibular groove with the presence of the neurovascular band during the 1st gestational trimester (10).

## TOPOGRAPHIC ANATOMY OF THE CLITORIS

The clitoris is an erectile organ homologous to the penis, responsible for the sensation of pleasure during female orgasm; knowledge of the anatomy of the clitoris is of fundamental importance in regenerative gynecology (2, 11). This organ is formed by erectile tissue, composed of the glans, the body (*corpora cavernosa*), and the vestibular bulbs. The glans and the bulb of the vestibule correspond to the corpus spongiosum. The clitoris is partially covered by structures derived from the labia minora – the prepuce and the frenulum of the clitoris. It is in contact with the supero-anterior portion of the pubic arch to which it is attached by the suspensory ligament of the clitoris (9, 11, 12). The labia minora are small mucous folds located between the labia majora, approximately 4 to 4.5 cm in length, with the space between them being called the vestibule of the vagina (13). Superiorly, the labia minora are related to the clitoris, dividing to form an upper portion that partially covers the glans, called the clitoral prepuce, and inferiorly the clitoral frenulum, which attaches to the lower portion of the glans (Figure-3). Inferiorly, the labia minora unite to form the labial frenulum or fork (13-15).

**Figure 3:**
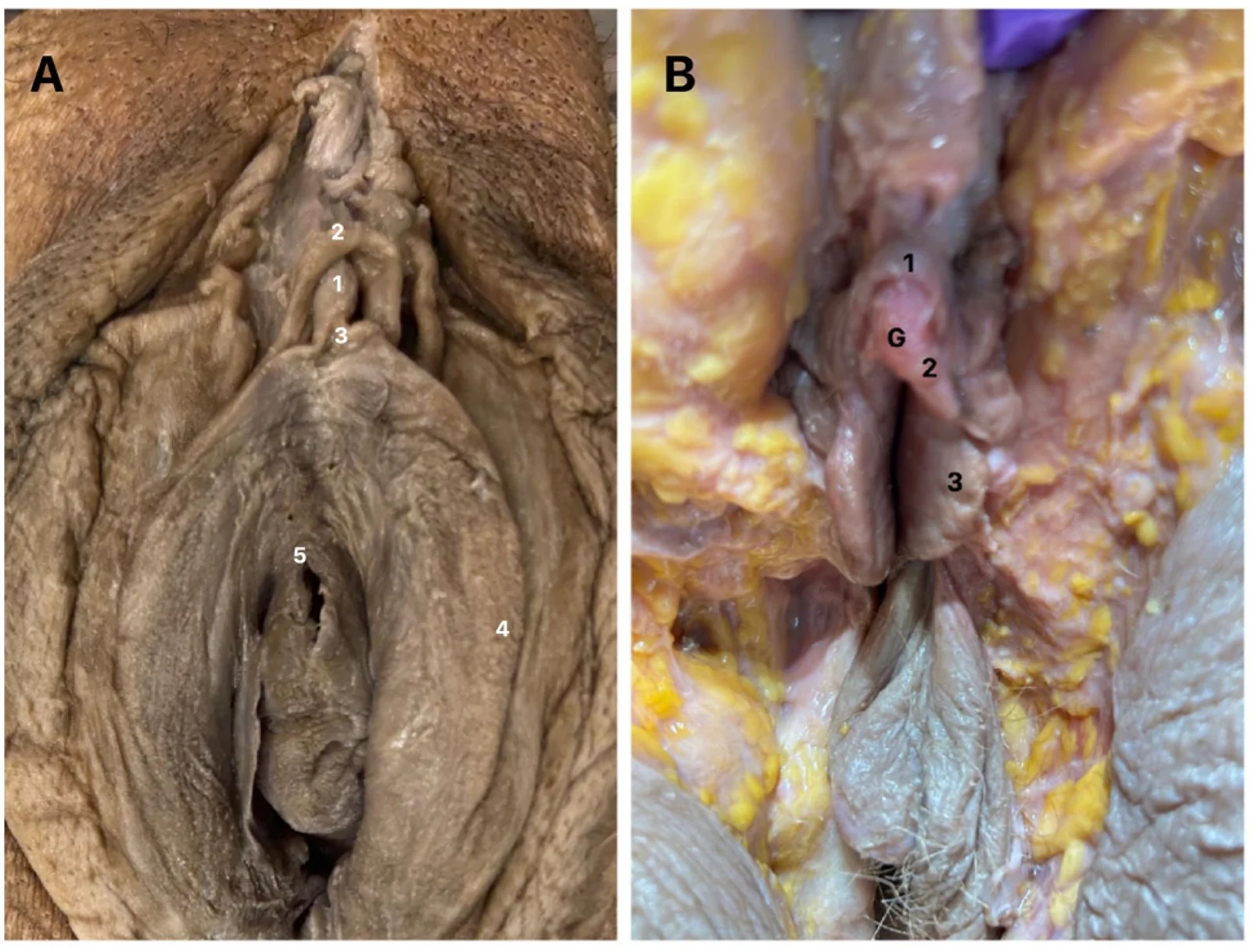
Glans of the clitoris.

A) The photo shows the perineal region of a formolized human corpus, we can observe: 1- clitoris glans, 2- clitoris hood, 3- clitoris frenulum, 4 – labia minora and 5 – urethra; B) Photograph of the perineal region of a fresh cadaver showing the relationship of the glans of the clitoris (G) with the labia minora (3), 1- Prepuce of the clitoris and 2- Frenulum of the clitoris.

The relationship between the clitoris and the labia minora is one aspect of importance of anatomical continuity and functional synergy. In gynecology, these structures are often viewed together as the clitoral-labial complex (16, 17). Understanding how they transition into one another is the most critical factor in achieving natural-looking and sensation-preserving results in aesthetic and reconstructive surgery. The labia minora do not simply end near the clitoris; they bifurcate (split) into two distinct cutaneous folds as they travel superiorly: Superior Fold (Prepuce or Hood): The upper portions of the labia minora fuse together over the clitoral glans to form the clitoral hood; Inferior Fold (Frenulum): The lower portions fuse beneath the clitoral glans, forming the frenulum, similar to the frenulum of the penis and the lateral connection: The lateral walls of the labia minora are tethered to the clitoral body. When the labia are pulled or stimulated, this tension is transmitted directly to the clitoral glans, facilitating the "gliding" mechanism during sexual activity (Figure-3) (16-20).

The clitoris has a shape similar to an inverted Y with the following portions: glans, body and crura (Figure-4). The glans is a protuberance that is partially exposed in the vulva, which has intimate contact with the clitoral body. The glans is the only externally visible portion, located at the superior junction of the labia minora. It is highly sensitive and covered by the clitoral hood (prepuce) (20-22). The clitoral body is located in the median plane immediately below the pubic arch and is composed of two *corpora cavernosa* covered by a tunica albuginea that sends a septum separating the *corpora cavernosa*. These are cylinders of erectile tissue that extend posteriorly and inferiorly (20-22). Each of the erectile bodies of the clitoris is attached to the ischiopubic ramus by a root, also called clitoral crura. These are the "legs" of the clitoris, extending along the pubic rami, a very important anatomical relationship for the interpretation of the image exams specially MRI (Figure-5).

**Figure 4:**
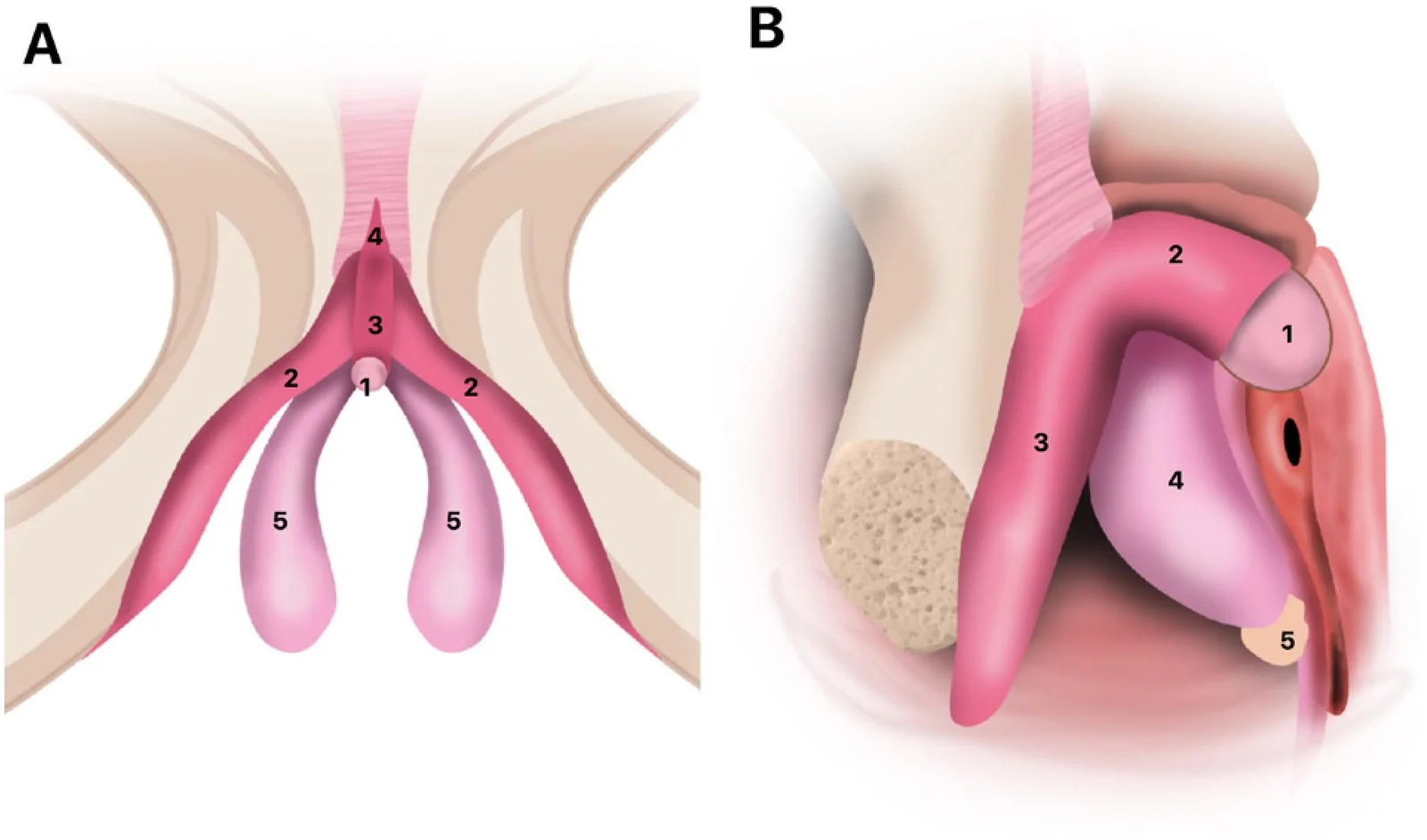
Anatomy of the Clitoris.

A) Schematic drawing of a frontal view of the clitoris and its relations with the pelvic bones. We can observe: 1- glans of the clitoris, 2 - crura of the clitoris, 3- body of the clitoris, 4 – suspensory ligament of the clitoris and 5 – bulb of the vestibule; B) Schematic drawing of a lateral view of the clitoris, we can observe: 1- glans of the clitoris, 2- body of the clitoris, 3 - crura of the clitoris, 4 – suspensory ligament of the clitoris and 5 – bulb of the vestibule.

**Figure 5:**
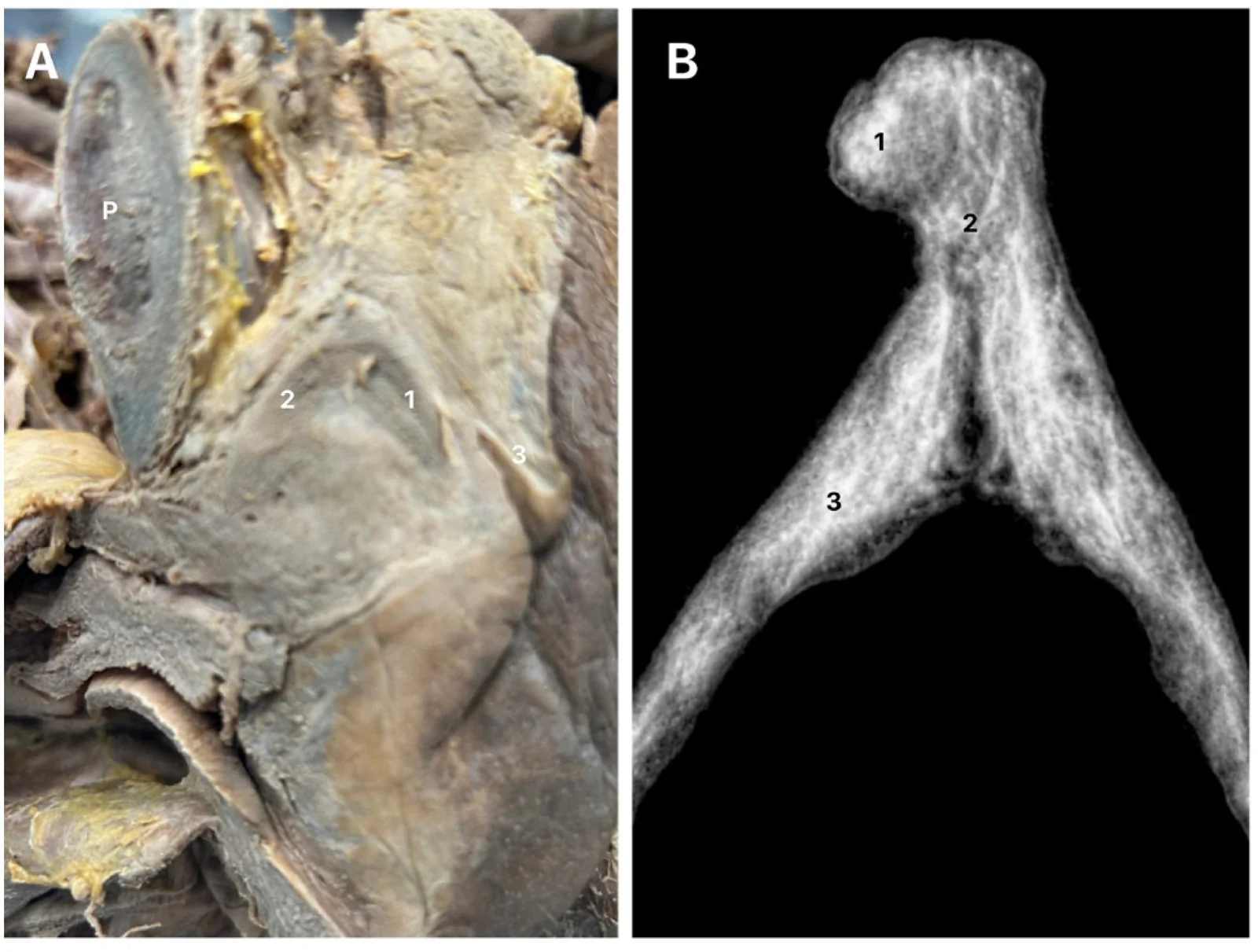
Sectional view of the clitoris. A) Sagittal section of a fresh frozen cadaver showing the shaft (1) and root (2) of the clitoris, p - pubis; B) A reconstruction of the clitoris showing the glans (1), shaft (2) and root (3).

A) Sagittal section of a fresh frozen cadaver showing the shaft (1) and root (2) of the clitoris, p - pubis; B) A reconstruction of the clitoris showing the glans (1), shaft (2) and root (3).

The vestibular bulb, homologous to the corpus spongiosum, is located below the bulbospongiosus muscle (Figure-6). The clitoris and the vestibular bulb are considered distinct erectile structures that nevertheless act together at the moment of female orgasm. Often considered the internal "wings," these masses of erectile tissue flank the vaginal opening and are embryologically homologous to the bulb of the penis (18-20).

**Figure 6:**
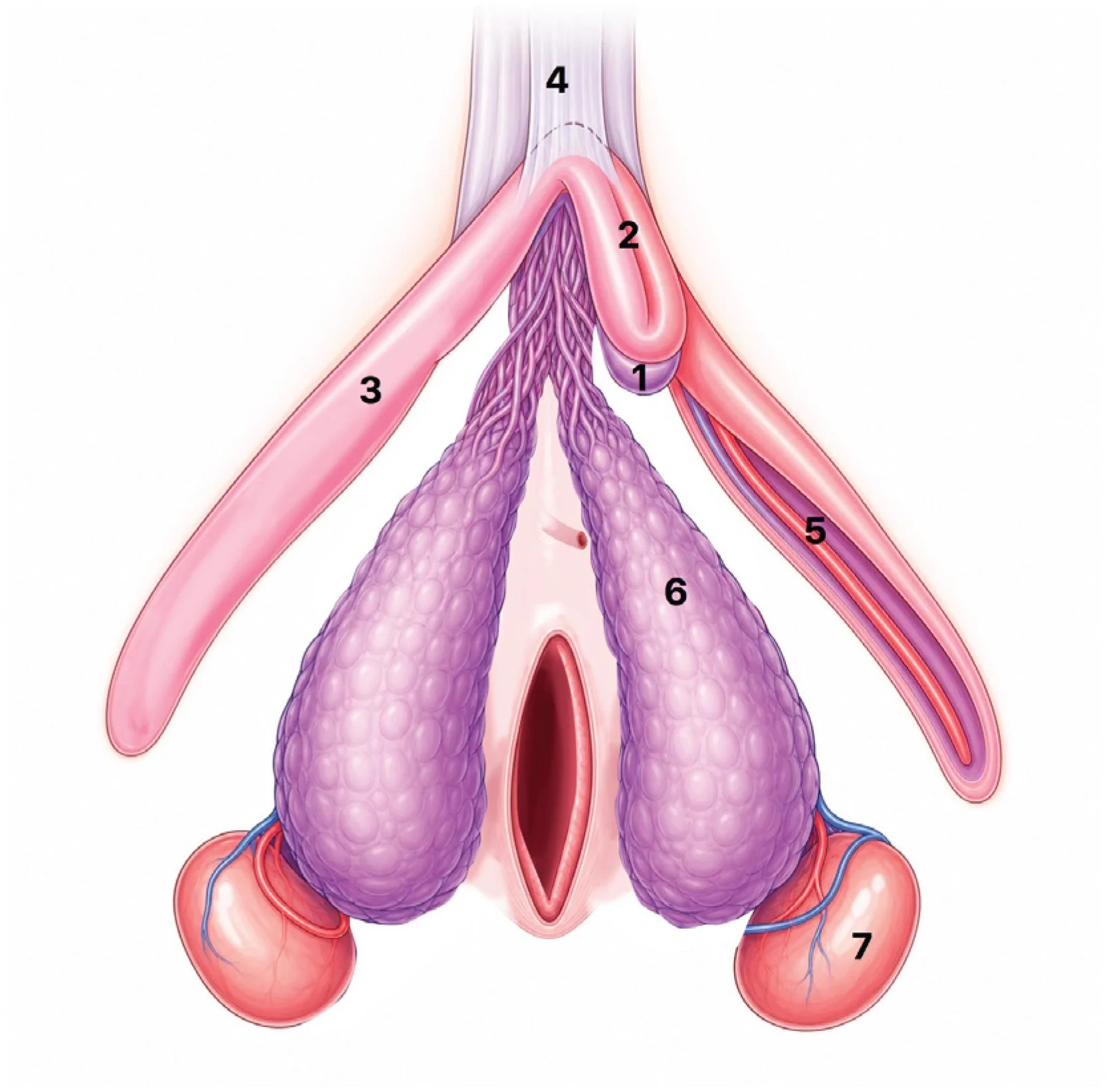
Clitoris vestibular bulb. Schematic drawing showing the most important clitoris structures. 1- Glans, 2- Shaft, 3- root, 4-suspensory ligament, 5- deep artery of the clitoris (cavernosa), 6- vestibular bulb and 7- Bartholin gland

Schematic drawing showing the most important clitoris structures. 1- Glans, 2- Shaft, 3- root, 4-suspensory ligament, 5- deep artery of the clitoris (cavernosa), 6- vestibular bulb and 7- Bartholin gland

## CLITORAL HOOD

Knowledge of the anatomy of the glans and clitoral hood is clinically important in cosmetic and reconstructive surgeries (23). The clitoral hood is formed by the superior fusion of the labia minora. The clitoral hood is a fold of skin that surrounds and protects the glans of the clitoris and is also called preputium clitoridis, clitoral prepuce, and clitoral foreskin (24). The upper (superior) folds fuse to form the prepuce (hood), while the lower (inferior) folds fuse beneath the glans to form the frenulum. The hood is composed of thin, highly elastic layer of skin and mucosal tissue (23, 24). It contains a high density of sebaceous glands that produce lubricating oils, preventing the sensitive glans from drying out or becoming irritated by friction. Some women have large clitoral hoods that completely cover the clitoral glans. Some of these can be retracted to expose the clitoral glans, such as for hygiene purposes or for pleasure; others do not retract. Other women have smaller hoods that do not cover the full length of the clitoral glans, leaving the clitoral glans exposed all the time (23).

The clitoral hood varies in size, shape, and thickness and usually has two distinct parts: a deep part - referred to as collar (present in more than 92% of cases) and a superficial part, referred to as hood (Figure-7) (25). Anatomical variations of the clitoris hood are frequent. The clitoris hood collar itself, especially the frenular part, could be continuous with the skin overlying the body of the clitoris, thus forming a pseudo-hood (Figure-7) (25). Zdilla in a very important paper described the clitoral hood anatomy and propose that the clitoral prepuce may exist as a unique structure distinct from the labia minora across the life cycle (25). The hood may join the crest of the labium minus, join at the lateral aspect of the labium minus, or not join at the labium minus at all (25).

**Figure 7:**
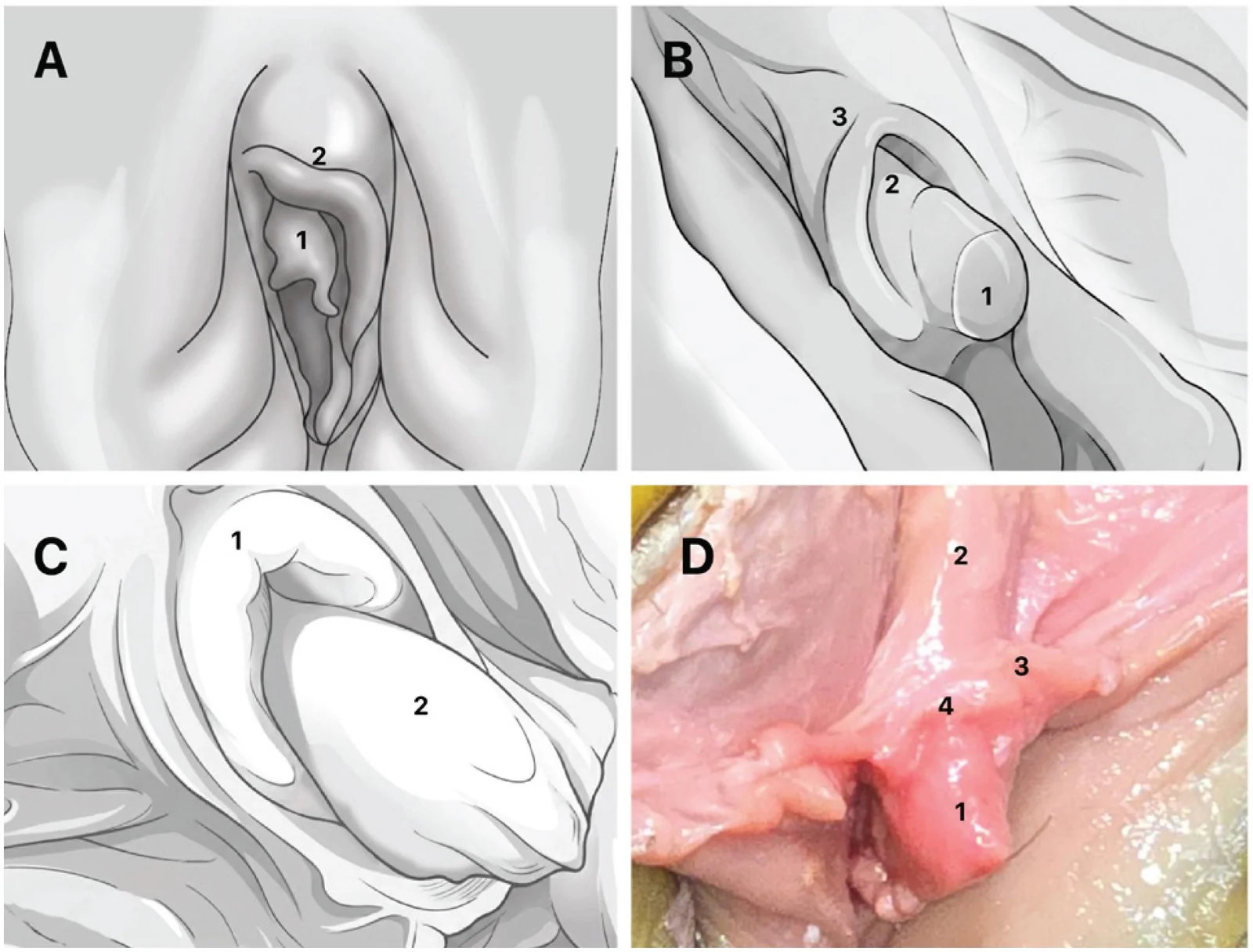
Clitoris Hood.

A) Schematic drawing showing the clitoris hood (2) and its relationship with clitoris glans (2); B) Schematic drawing showing the hood retracted (3) to expose the clitoral glans (1) and shaft (3); C) Schematic drawing showing another case of hood retraction (1) with the exposition of the clitoris glans (2) and D) Fresh frozen cadaver with dissection of hood (3) with the glans (1) and clitoris shaft (2) exposition, we can observe the hood deep part - the collar (4).

Clitoral hood is a frequent focus in aesthetic and functional gynecology. Some women experience "clitoral phimosis" or have redundant (excess) hood tissue that may interfere with hygiene or sexual sensation (26). The presence of clitoral adhesion and phimosis can trap smegma, causing pain (clitorodynia) or a significant decrease in sexual pleasure because the "gliding" mechanism is lost. Treatment involves manually or surgically releasing these adhesions to restore mobility (26).

Hood innervation has a great importance during surgical procedures. The dorsal nerves of the clitoris run just beneath the deep fascia of the clitoral body. Surgeons performing a hoodectomy must remain strictly in the superficial subcutaneous plane to avoid permanent desensitization (27, 28). Often performed alongside a labiaplasty, the goal is to create a proportional appearance where the glans is visible but still protected. Sensory Mapping: High-resolution scans show that sensory branches of the pudendal nerve extend into the preputial tissue (27, 28). While not as sensitive as the glans, the hood is a legitimate erogenous zone. Over-resection during surgery can lead to a loss of the "pre-orgasmic" build-up provided by hood movement (27-29).

## CLITORIS GLANS

The glans clitoris is the most visible and highly sensitive part of the clitoral complex. While historically often mistaken for the entirety of the clitoris, it represents the distal termination of the clitoral body (corpus) and serves as the primary epicenter for sexual sensation in the female body (Figure-3). The glans is a small, rounded structure, typically measuring 3 to 6 mm in diameter, though there is significant natural anatomical variation (16, 30). It is situated at the anterior junction of the labia minora, partially or fully covered by the clitoral hood. Unlike the clitoral body and crura, which are composed of cavernous erectile tissue, the glans is primarily made of highly vascularized connective tissue and an extremely dense concentration of nerve endings.

Glans effectively is a sensory cap for the erectile bodies (31, 32). It is connected to the clitoral body that engorges with blood during arousal; the glans is pushed forward and becomes more prominent (27, 28). The glans is the destination for the terminal branches of the dorsal nerves of the clitoris (DNC) (32). According to the landmark 2026 synchrotron mapping studies, the glans contains over 10,000 sensory nerve fibers (27, 28, 32). This density is roughly 15 times higher than that of the glans penis relative to its size. The 2026 data revealed that the DNC branches into a tree-like network within the glans, ensuring that even the slightest touch triggers a significant neurological response (27, 28, 32). The glans has important sensory receptors that are packed with specialized receptors, specifically Meissner's corpuscles (light touch) and Pacini corpuscles (vibration), which are tuned to the frequencies often used in manual or mechanical stimulation.

During the female sexual response cycle, the glans undergoes distinct changes (29, 31, 32): Engorgement: While the glans does not contain the same large blood-filled spaces as the *corpora cavernosa*, it becomes tumescent (swollen) due to increased blood flow in its dense capillary network. Retraction: Interestingly, as a woman nears orgasm, the glans often retracts under the clitoral hood. This is a protective mechanism; the glans becomes so sensitive that direct, high-intensity contact may become painful or overwhelming. Sensitivity Peaks: The glans acts as the "trigger" for the somatic reflex arc that leads to the rhythmic pelvic floor contractions of an orgasm (29, 31).

During gynecological surgery the glands need to be preserved because of its extreme sensitivity and critical role in pleasure (30, 33). In procedures like clitoral hood reduction, the surgeon must ensure that no incisions or sutures interfere with the blood supply to the glans or the terminal nerve branches entering it. In some cases, the prepuce can become adhered to the glans (clitoral phimosis) due to inflammation or hygiene issues, which can trap debris (smegma) and lead to pain or decreased sensation (13, 14, 26). In the field of aesthetic and reconstructive gynecology, surgical techniques involving the clitoris focus on two main objectives: improving anatomical harmony (aesthetic) and restoring function or alleviating pain (reconstructive) (13, 14, 26). A common mistake in aesthetic surgery is performing a significant labiaplasty without addressing the clitoral hood. This can lead to a "clitoral unmasking" or a top-heavy appearance. If the labia minora are resected too aggressively near the clitoris, the natural "tugging" sensation that contributes to arousal may be lost. High-quality aesthetic surgery aims to preserve the frenulum (the inferior connection), as this area is rich in sensory receptors and maintains the structural integrity of the clitoral-labial unit.

In some cultures, female genital mutilation (FGM) is practiced as a rite of passage into womanhood, is perceived as an improvement to the appearance of the genitalia or is used to suppress or reduce female sexual desire and pleasure (including masturbation). In cases of genital mutilation reconstruction, the "pull-down" technique aims to bring the buried clitoral body to the surface to create a "neo-glans" that can once again interface with external stimuli (34). If the labia minora have been partially preserved, these flaps of skin are often used to reconstruct a new clitoral hood (prepuce) over the newly transposed clitoral stump (35). The goal is to re-create the "hood-glans" interface, allowing for the protective and stimulatory gliding motion to occur once again (34-36).

## CLITORIS HISTOLOGY

Histology of the human clitoris reveals a highly specialized organization of erectile tissue and neurovascular structures, similar in several aspects to the penis, but with functions exclusively related to sexual pleasure. The clitoris is composed of three main parts: glans, body (or shaft), and crura (37, 38). Histologically, these regions are formed mainly by erectile tissue of the corpus cavernosum type, surrounded by dense connective tissue (2). The erectile tissue of the clitoris consists of vascular spaces (sinusoids) lined by endothelium and trabeculae of connective tissue containing collagen fibers, elastic fibers, and smooth muscle. These elements allow blood engorgement during arousal, promoting clitoral erection. Unlike the penis, the clitoris does not have a corpus spongiosum or associated urethra, being formed exclusively of cavernous tissue.

The clitoral glans is lined by stratified squamous epithelium, which can vary from keratinized to non-keratinized depending on the region (Figure-8). Rich sensory innervation is seen just below the epithelium (37, 38). The clitoris presents: Helicine arteries, responsible for filling the sinusoids, venous network that regulates blood flow during tumescence and detumescence (2). The erectile tissue is enveloped by a tunica albuginea, composed of dense connective tissue rich in collagen, which provides structural support and aids in maintaining intracavernous pressure during erection. One of the most striking aspects of clitoral histology is its extreme density of nerve endings: Abundant presence of Meissner's corpuscles (tactile sensitivity), Pacinian corpuscles (pressure and vibration) and free nerve endings. This organization explains the high sensitivity of the clitoral glans.

**Figure 8:**
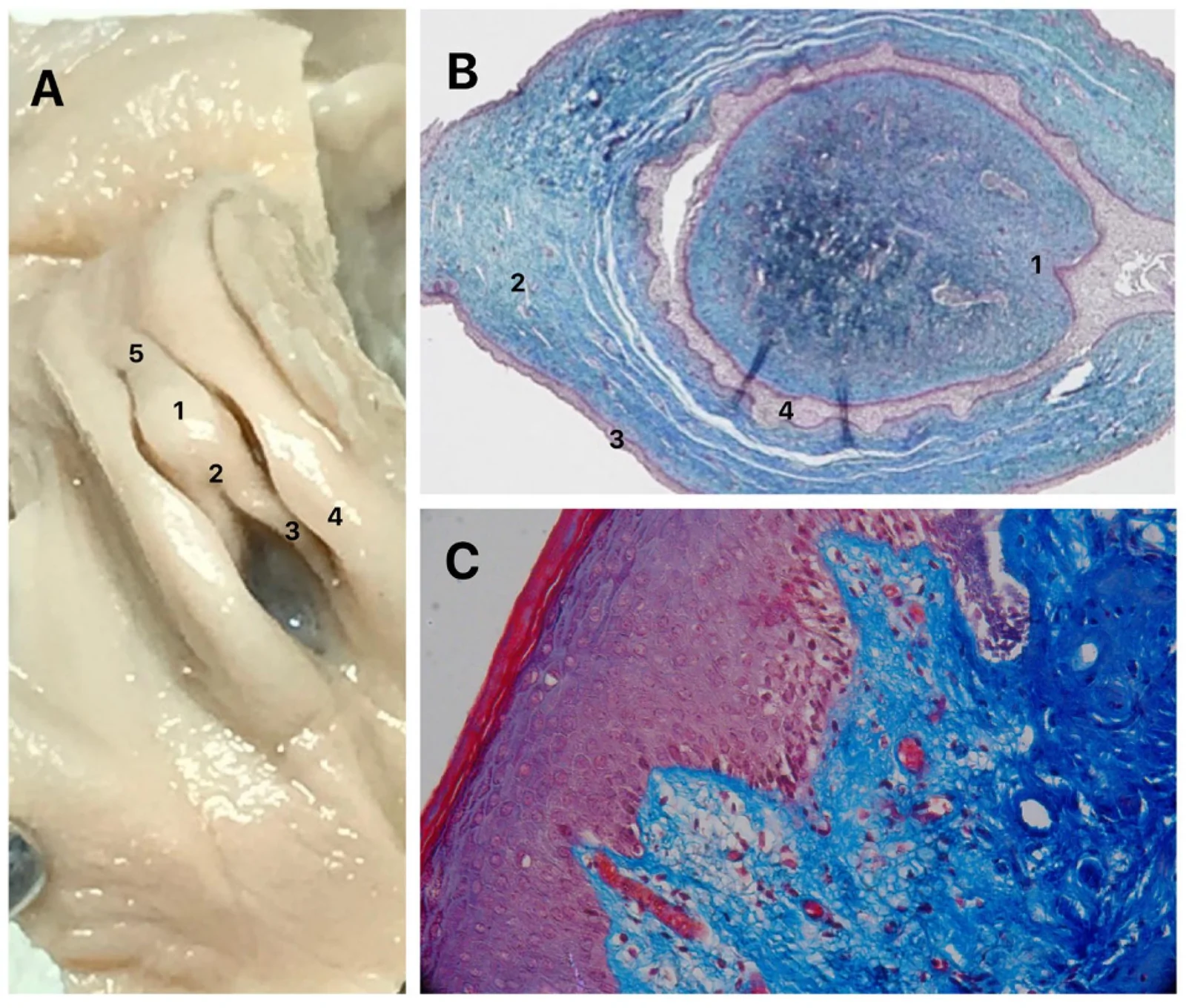
Clitoris Histology.

A) The figure shows the clitoris of a fetuses with 16 weeks post-conception, we can observe the clitoris glans (1), clitoris frenulum (2), labia menorah (3), labia majora (4) and clitoris shaft (5); B) Photomicrography of a female human fetus with 17 weeks post-conception. We can observe the histology arrangement in clitoris, 1- clitoris glans, 2 – clitoris hood, 3- epithelium of the clitoris and 4 – preputial lamella of clitoris, Masson Trichrome X40. B) The figure shows a photomicrography of clitoris hood, and we can observe the clitoris epithelial height measurement. Masson Trichrome X400.

## CLITORIS SUSPENSORY LIGAMENT

The suspensory ligament of the clitoris is a condensation of connective tissue and deep fascia that connects the clitoral body to the pubic symphysis and anterior pelvic structures. It supports and stabilizes the clitoris, transmits tension during erection, and is related to the deep dorsal vein, arteries and neurovascular bundles (37, 38). It is often described as having superficial and deep components that merge with the suspensory apparatus of the mons pubis and the deep fascia of the urogenital diaphragm. Knowledge of its anatomy is relevant in genital surgery (cosmetic and reconstructive), pelvic trauma and pelvic floor dysfunction.

The suspensory ligament is located in midline, extending from the dorsum of the clitoral body to the pubic symphysis/retro-pubic region (37, 38) (Figure-9). It lies deep to the mons pubis, continuous with deep fascia, intimately associated with the deep dorsal vein and dorsal nerves of the clitoris. It is described as fibrous band(s) with variable thickness; authors report superficial and deep laminae or a single suspensory plate depending on dissection technique and specimens and has the function of mechanical support of the clitoral body; contributes to positional stability and may influence biomechanical transmission during sexual arousal (37, 38). The suspensory ligament is an important landmark in female genital aesthetic and reconstructive procedures (labiaplasty, clitoral hood reduction, suspensory release) and may be considered in evaluation of pelvic pain after trauma surgery.

**Figure 9:**
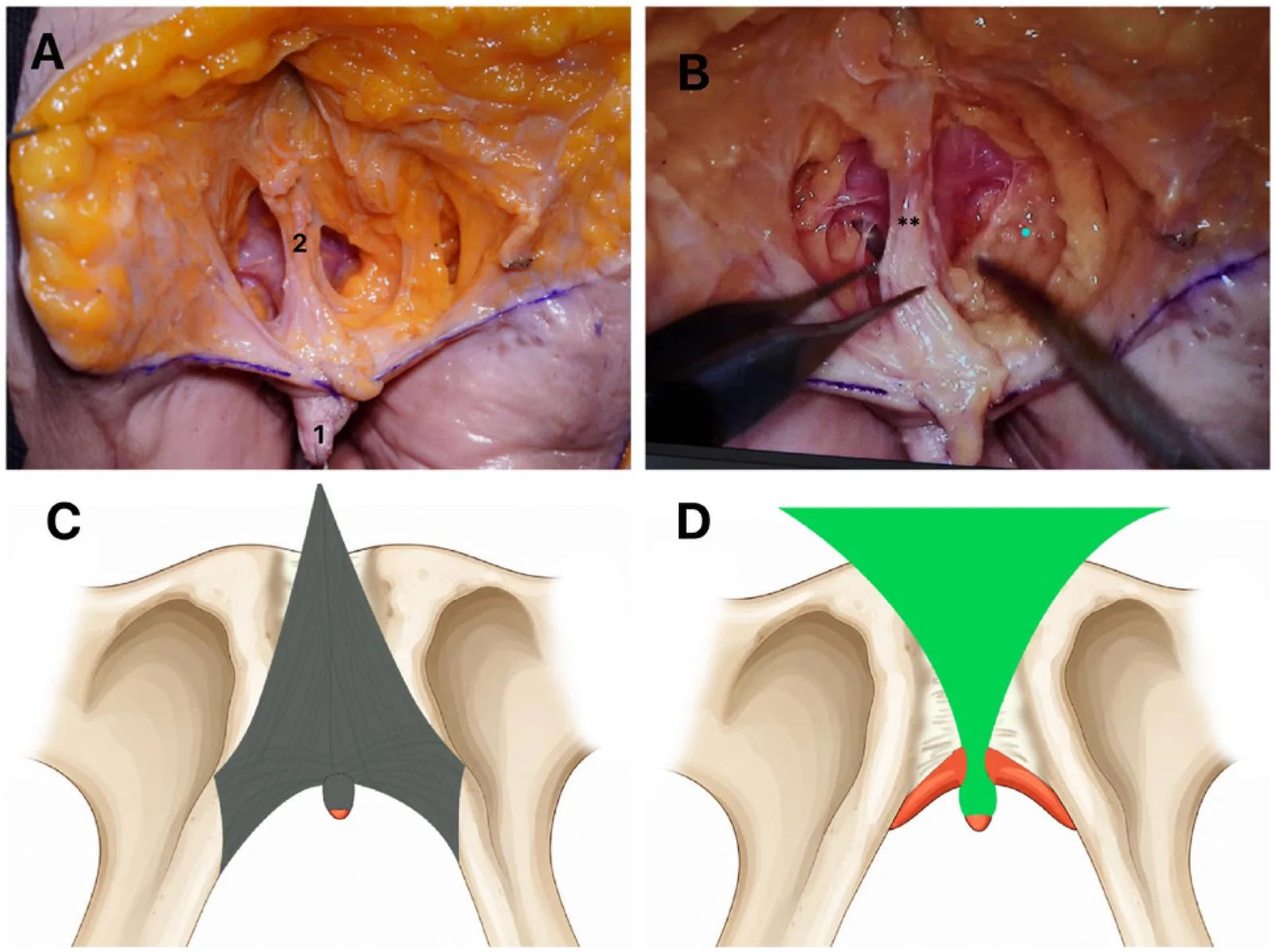
Clitoral suspensory ligament.

A) Photograph of the perineal region of a fresh-frozen cadaver showing the clitoral suspensory ligament (2), 1 – clitoris glans; B) Photograph of the same fresh-frozen cadaver showing the suspensory ligament after dissection; C) Schematic drawing showing the superficial component of the clitoris suspensory ligament (Black). This superficial component arises from the anterior abdominal wall, courses over the pubic symphysis without full fixation to it, envelops the clitoral body, and gives off lateral extensions resembling airplane wings, which traverse the labia majora and insert into the fascia lata in the ipsilateral inguinal region, we can observe the clitoris glans in orange and D) Schematic drawing showing the Intermediate component of clitoris suspensory ligament (green). This component is located in midline, extending from the dorsum of the clitoral body to the public symphysis/retro-pubic region. This component completely encloses the clitoral body and sends lateral extensions to the labia majora. We can observe the clitoris glans and clitoris root in orange; the clitoris shaft is not observed because the intermediate component involves this portion.

A relevant anatomical-histological study introduced a new three-dimensional interpretation of the clitoral suspensory ligament by describing three components (superficial, intermediate, and deep) of which the intermediate component appears to be functionally the most relevant (39) (Figure-9). It arises from the anterior abdominal wall, courses over the pubic symphysis without full fixation to it, envelops the clitoral body, and gives off lateral extensions resembling airplane wings, which traverse the labia majora and insert into the fascia lata in the ipsilateral inguinal region. Because this component is composed of dense connective tissue rich in collagen fibers, it plausibly contributes to the barrier effect suggested by the findings of this study (39).

## CLITORIS BODY AND BULBUS

The clitoral body (shaft) lies beneath the skin, extending inward from the glans. The shaft is composed of two *corpora cavernosa* (erectile tissues), similar in structure to the penis, surrounded by a fibrous sheath called the tunica albuginea, separated by an incomplete septum. The septum is an extension of the tunica albuginea, which is a fibrous connective tissue sheath that surrounds the *corpora cavernosa* superficially (Figure-10) (37, 38). The body bifurcates laterally into the left and right crura that follow the inferior border of the ischiopubic rami and are located deep to the respective ischiocavernosus muscles (40). During arousal, it fills with blood and becomes engorged, contributing to increased sensitivity and rigidity; and it connects the external glans to the internal crura. Functionally, the body acts as a central conduit of erectile tissue, transmitting pressure and stimulation from internal movement to the glans (2).

**Figure 10:**
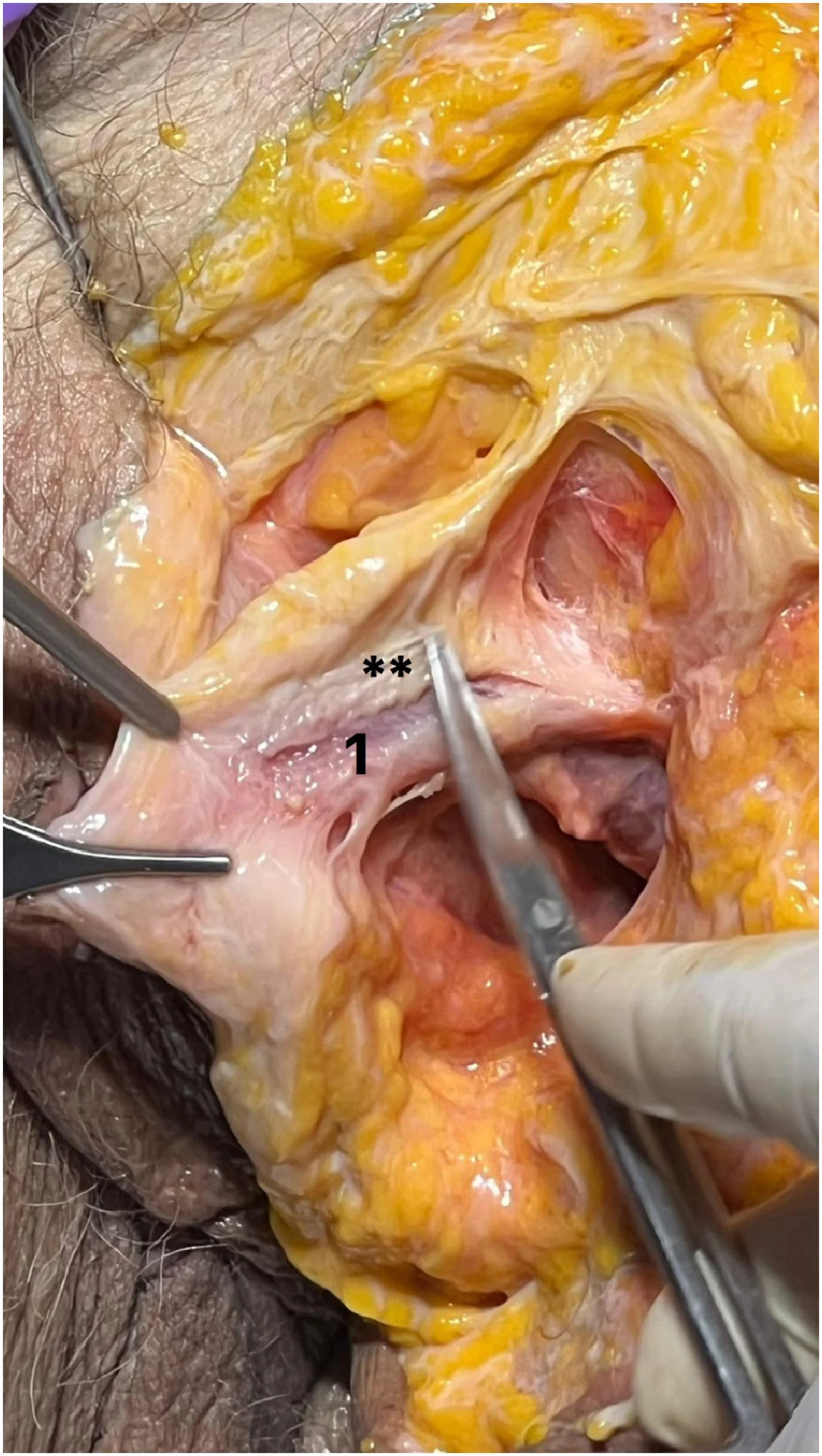
Clitoris shaft with albuginea.

Photography of a dissection in female fresh frozen cadaver showing the clitoris shaft after the dissection of the tunica albuginea (1). We can observe the neurovascular bundle in clitoris dorsal portion (**).

The vestibular bulbs are often overlooked but are key to sexual function and are composed of erectile tissue (Figure-5). They are located on either side of the vaginal opening, beneath the labia and made of spongy erectile tissue (similar to corpus spongiosum in males). They swell during arousal, narrowing the vaginal opening and increasing friction and are connected to the clitoral body via vascular and neural networks and maintain a consistent relationship with the clitoral complex and can completely or partially fill the space between labia minora, body, and crura (2). These bulbs are partly responsible for the sensation of fullness and pressure during arousal and penetration.

Measurements of the clitoris vary a lot from person to person, and they can also change depending on factors like arousal, age, and hormonal influences. Therefore, anatomical studies give us useful average ranges for the glans and shaft (body). A recent metanalysis presented important information about the clitoris measures (40). The average length and width of the glans was 6.40 mm (range 1.00-21.00 mm) and 5.14 mm (range 2.00-32.00 mm), respectively. The average length and width of the body was 25.46 mm (range 5.00–59.00 mm) and 9.00 mm (range 5.00-20.00 mm), respectively. The average length and width of the crura was 52.41 mm (range 23.00–90.00 mm) and 8.71 mm (range 2.00–13.00 mm), respectively. The average length and width of the bulb was 52.00 mm (range 13.00–70.00 mm) and 10.33 mm (3.00–29.00 mm), respectively (40). The authors of this metanalysis concluded that the clitoral components vary in size and shape, whilst remaining within the normal range (40).

## CLITORIS VESSELS

Knowledge of the vascularization and innervation of the clitoris has great application in surgical procedures in regenerative gynecology. The clitoris and labia minora share more than just skin; they share a blood and nerve supply derived from the same source. Both receive blood from branches of the internal pudendal artery. Specifically, the labial arteries and the dorsal artery of the clitoris arise from the same perineal trunk.

The blood supply to the clitoris is primarily derived from the internal pudendal artery, which is a branch of the internal iliac artery (Figure-11). The pudendal artery originates 3 arteries which has the clitoris as its irrigation area (Figure-12): 1) Dorsal artery of the clitoris, that runs along the dorsal surface of the clitoral body, deep to the Buck's fascia. It provides the primary blood supply to the glans and the overlying prepuce (hood); 2) Deep artery of the clitoris, that runs through the center of the *corpora cavernosa* and is essential for tumescence (erection) and 3) Bulbar artery that supplies the vestibular bulbs and the surrounding erectile tissue.

**Figure 11:**
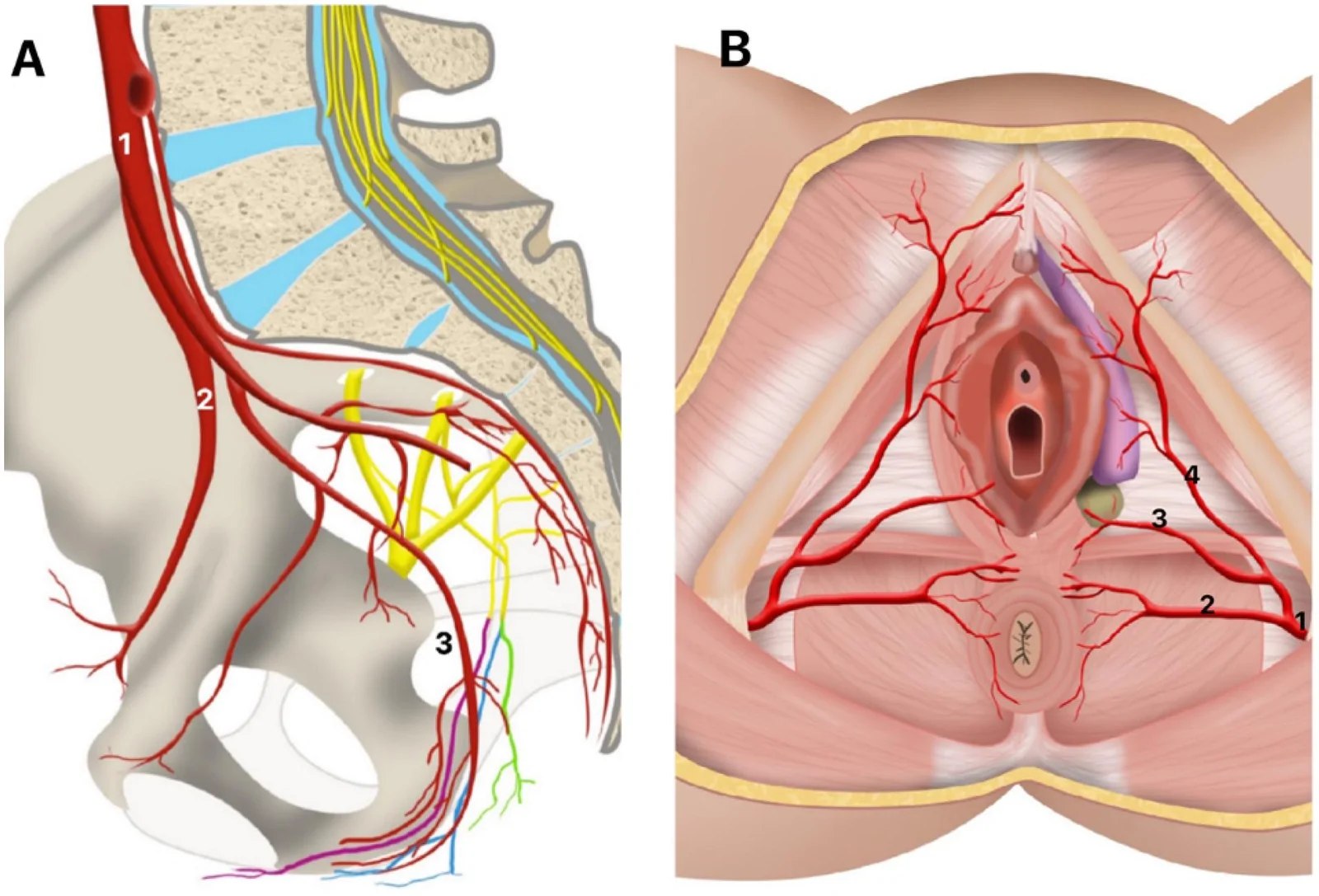
Internal Pudendal artery distribution.

A) Schematic drawing of the vessels responsible for the vascularization of the pelvic viscera in women in a lateral view, 1- Internal iliac artery, 2 - internal iliac artery and 3 – internal pudendal artery, branch of the anterior trunk of the internal iliac artery and B) Schematic drawing showing the branching of the internal pudendal artery (1), 2- inferior rectal branch, 3- transverse perineal branch and 4 - perineal branch.

**Figure 12:**
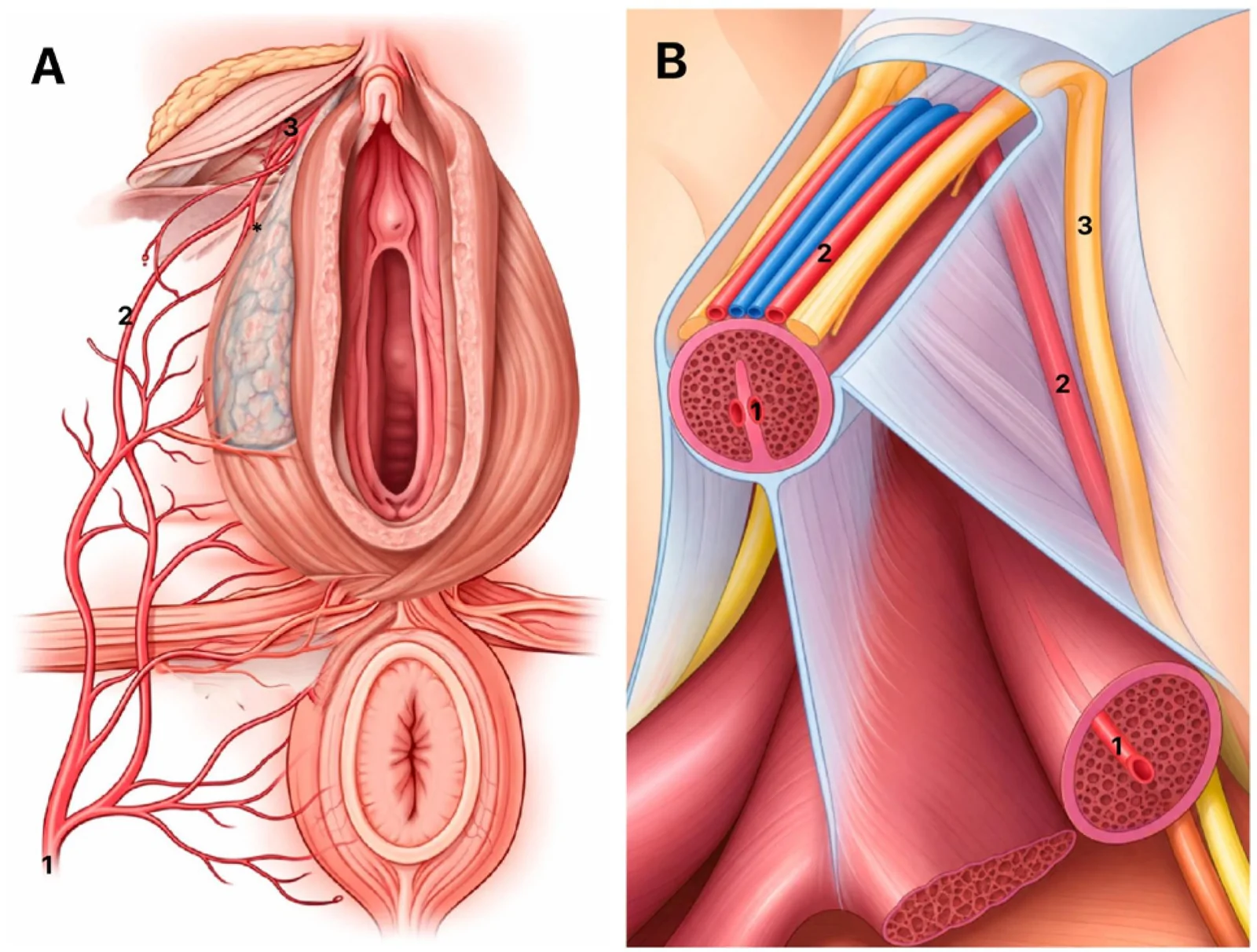
Clitoris Arteries.

A) Schematic drawing showing the pudendal internal artery (1) distribution. The clitoris arteries origin from the perineal artery (2). We can observer the dorsal clitoris artery (3) and clitoris deep artery origin (*) and B) Schematic drawing of the clitoris arteries: The clitoris deep artery (1) penetrates the root of the clitoris and follows a path through the central region of the entire clitoris, including the body, where it can also be called the cavernous artery of the clitoris (1), the superficial clitoral artery (2) is a branch of the perineal artery and runs closely and superficially to the tunica albuginea of the clitoris. It is one of the elements that constitute the neurovascular bundle in the dorsal region of the clitoris and is of great surgical importance. The dorsal nerve of the clitoris (3) is also observed in this figure.

In aesthetic surgery, particularly during a clitoral hoodectomy, surgeons must remain superficial to the deep fascia to avoid compromising these terminal branches, which could lead to ischemia of the glans. Venous drainage mirrors the arterial supply, primarily through the dorsal vein of the clitoris, which eventually drains into the vesical or vaginal venous plexuses. These structures, together with the dorsal nerve of the clitoris, which will be described in the next topic, form a neurovascular bundle located in the dorsal region of the clitoris (Figure-13). The dorsal region of the clitoris is therefore a region of great importance during surgeries of this organ (9, 41). Preservation of the clitoral bundle is a key point in surgical interventions such as clitoroplasty or clitoropexy.

**Figure 13:**
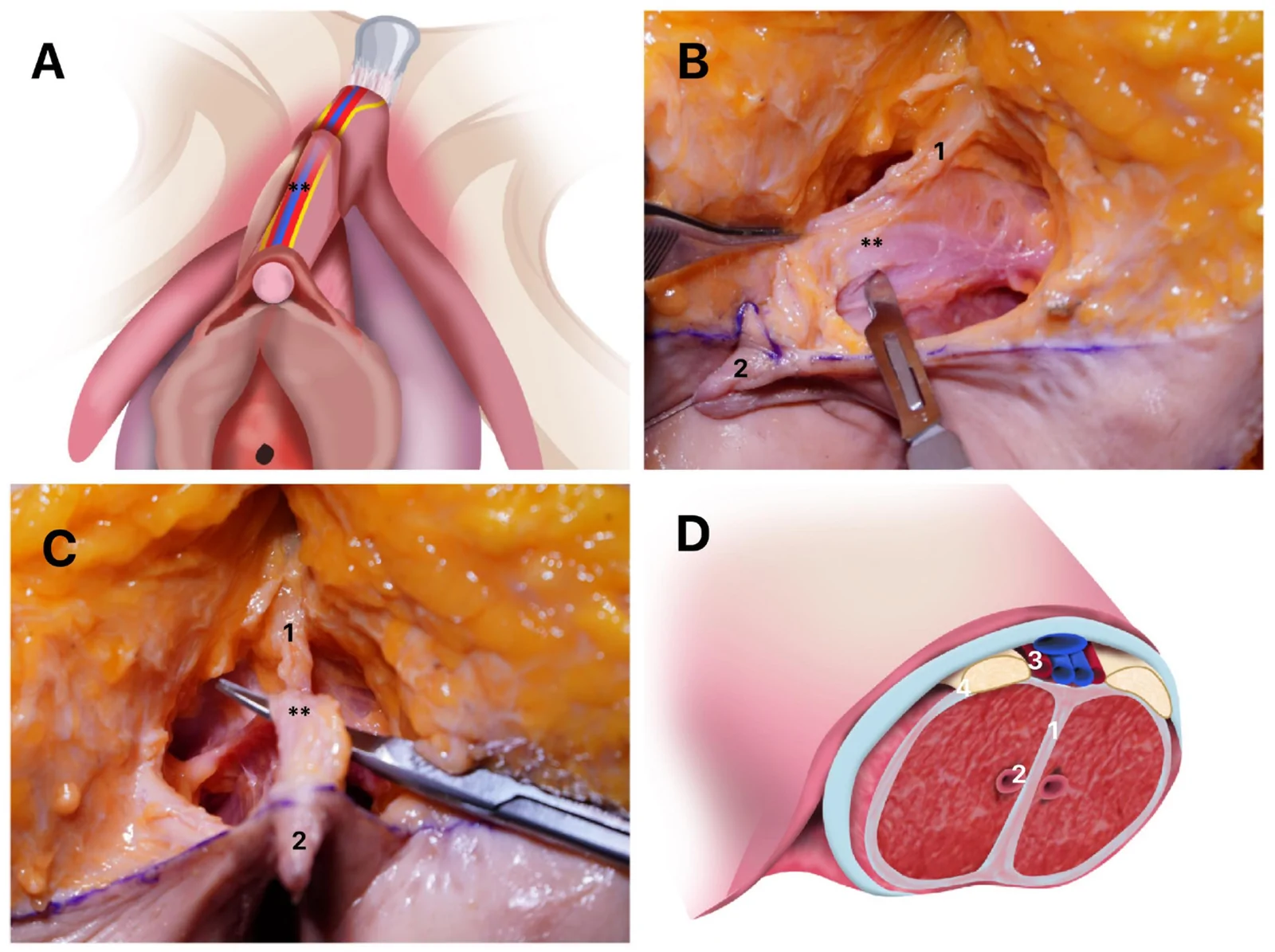
Clitoral neurovascular bundle.

A) Schematic drawing showing the relationship of the clitoral neurovascular bundle (**) with the region of the clitoral body (3); B) The figure shows a dissection in a fresh frozen cadaver of the clitoral neurovascular bundle (**) and its relation to the dorsal region of the clitoral body, 1 – suspensory ligament of clitoris, 2- clitoris glans; C) The figure shows in the same fresh frozen cadaver the individualization of the clitoral neurovascular bundle (**), 1 – suspensory ligament of clitoris, 2- clitoris glans and D) Schematic drawing of a transverse section of the clitoral body showing the topography of its components: 1- Septum between the erectile (cavernous) bodies of the clitoris, 2- deep artery of the clitoris, 3- dorsal artery of the clitoris, 4- dorsal nerve of the clitoris.

## CLITORIS NERVES

Clitoris is one of the most densely innervated structures in the human body, specifically designed for sensory reception. While the sensory innervation of the clitoris (via the pudendal nerve) is responsible for the "feeling" of touch, the autonomic innervation is the "engine" that drives the physiological changes of sexual arousal, such as vasocongestion and tumescence. This system operates largely outside of conscious control, through the complex interaction of the sympathetic and parasympathetic pathways (42).

The pudendal nerve is the primary source of somatic sensation. Specifically, the dorsal nerve of the clitoris (the terminal branch of the pudendal nerve) travels along the clitoral body to reach the glans. This nerve provides the high-resolution tactile sensitivity required for sexual arousal (28, 32, 42). The clitoris also receives autonomic fibers from the cavernous nerves, which originate from the uterovaginal plexus (Frankenhäuser's ganglion). The nerve travels along the ischiopubic ramus and emerges beneath the pubic symphysis. It runs along the dorsal aspect of the clitoral body, typically located at the 11 o’clock and 1 o’clock positions. These nerves are highly sensitive and relatively superficial near the glans (32, 43). During labiaplasty (especially the "wedge" technique) or hood reduction, incisions made too deeply or too medially toward the midline of the dorsal body can result in permanent desensitization (anorgasmia) or the formation of painful neuromas (Figure-14).

**Figure 14:**
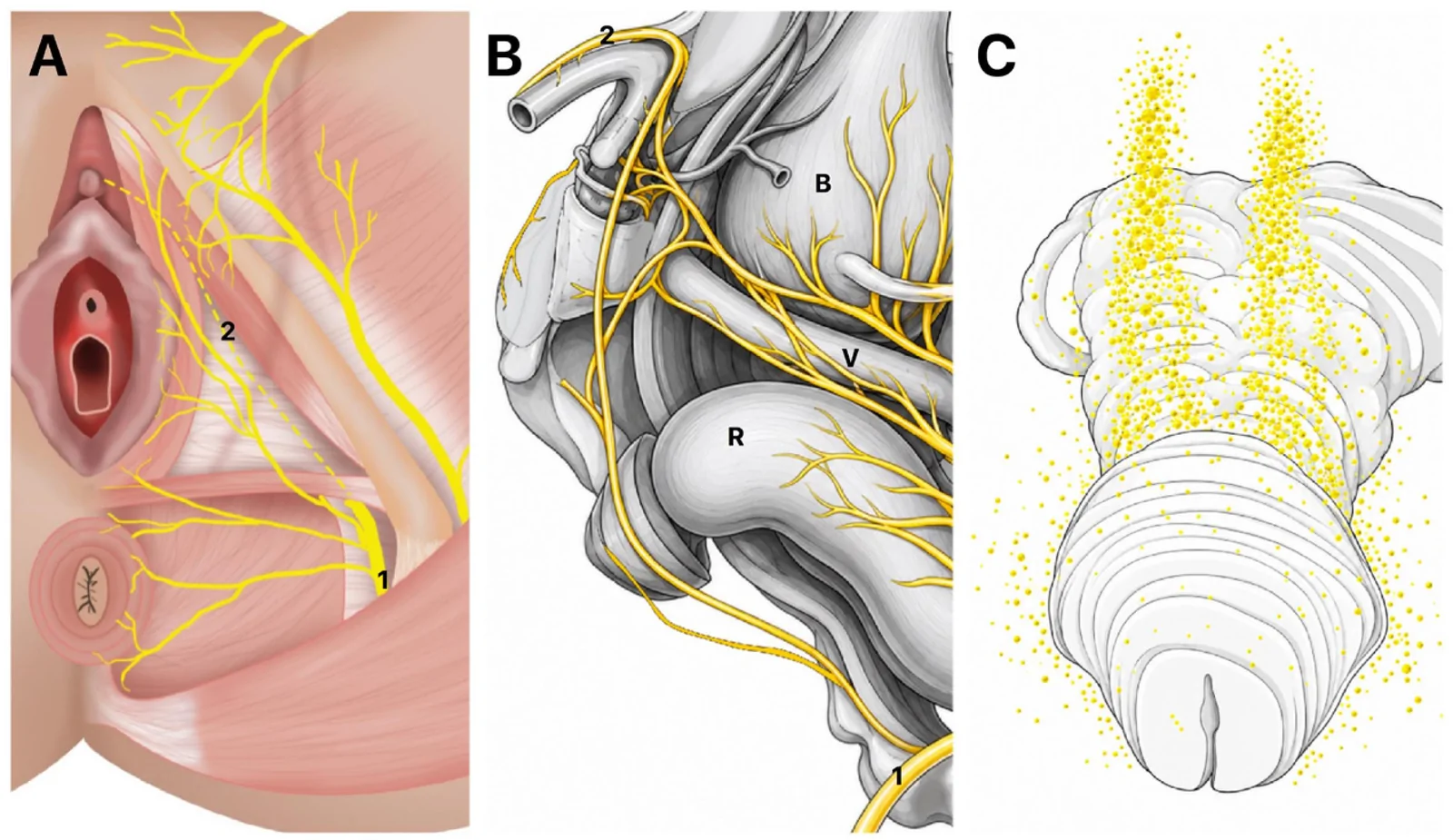
Somatic Innervation of Clitoris.

A) Schematic drawing showing the innervation of the female perineum: 1- pudendal nerve, 2 - dorsal Nerve of the Clitoris (dotted line) originates from the perineal branch; B) Schematic drawing showing the pudendal nerve (1) originating the dorsal nerve of the clitoris (2) and its path in perineum, B- bladder, V- vagina, R- rectum and C) Schematic drawing showing the dorsal nerve of clitoris distribution (yellow) in clitoris shaft and glans.

The somatic innervation of the clitoris is responsible for the transmission of acute sensory information—touch, pressure, temperature, and, most importantly, erogenous pleasure—to the central nervous system. Unlike the autonomic system, which handles the "involuntary" vascular response, the somatic system provides the "voluntary" and conscious perception of stimulation. The clitoris receives its somatic sensory supply almost exclusively from the pudendal nerve, which originates from the ventral rami of the S2, S3, and S4 sacral spinal nerves.

As the pudendal nerve travels through the pelvis, it enters Alcock's canal (pudendal canal) and eventually divides into three terminal branches: The inferior rectal nerve, The perineal nerve and The Dorsal Nerve of the Clitoris (DNC) (Figure-14). The DNC is the most critical branch for sexual function. It is a purely sensory nerve (in some models, it carries minor sympathetic fibers, but its primary role is somatic) that travels forward along the ischiopubic ramus.

The DNC emerges from beneath the pubic symphysis and enters the clitoral complex at the level of the suspensory ligament. It runs along the dorsal (top) surface of the clitoral body, deep to the Buck's fascia but superficial to the tunica albuginea of the *corpora cavernosa*. Classically, the two bilateral nerves are described as running at these specific "clock" positions along the clitoral shaft (Figure-14). The Glans "Tree": As confirmed by 2026 synchrotron mapping, once the DNC reaches the glans, it does not stop; it branches into an extremely dense network of terminal fibers. This "nerve tree" ensures that every micron of the glans is highly sensitive (28, 29, 32).

The somatic system in the clitoris is unique due to the concentration of specialized mechanoreceptors: Pacini Corpuscles: Sensitive to vibration and deep pressure; Meissner Corpuscles: Sensitive to light touch; Free Nerve Endings: Responsible for temperature and pain (nociception). With over 10,000 sensory axons (28, 29, 32), the clitoris has the highest density of these receptors in the human body. This allows for the phenomenon of spatial summation, where stimulation across the surface of the glans rapidly builds to the threshold required for a somatic reflex—the orgasm.

The Parasympathetic fibers has a function of trigger the relaxation of smooth muscles in the erectile tissue, allowing for increased blood flow and engorgement. The Sympathetic fibers are involved in the resolution phase and smooth muscle contraction. The sensory innervation of the clitoris is provided by the dorsal nerve of the clitoris, the terminal branch of the pudendal nerve (27, 32, 43). The autonomic fibers that reach the clitoris originate from the inferior hypogastric plexus (also known as the pelvic plexus). This plexus is a dense network of nerves located on the lateral walls of the rectum and vagina (Figure-15). The parasympathetic input arises from the pelvic splanchnic nerves (nerve roots S2 through S4). The sympathetic input arises from the hypogastric nerves (nerve roots T10 through L2 (27, 32, 43).

**Figure 15:**
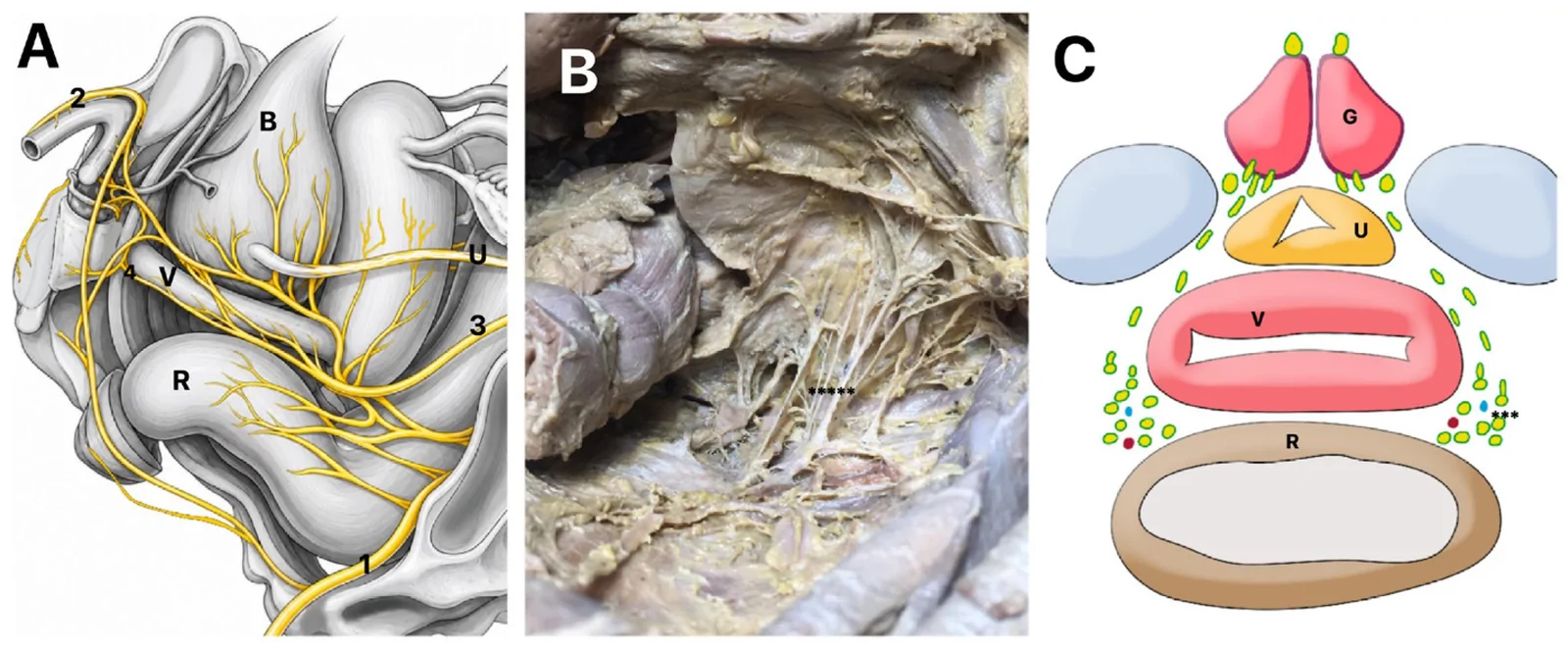
Autonomic innervation of clitoris.

A) Schematic drawing showing the autonomic innervation of the female perineum: 1- pudendal nerve, 2 - dorsal Nerve of the Clitoris, 3- Left hypogastric nerve, 4 – origin of cavernous nerve of clitoris, B- bladder, V- vagina, R- rectum; B) Dissection of fixed female pelvis showing the inferior hypogastric plexus distribution (***) and C) Schematic drawing showing the autonomic innervation of clitoris distribution (yellow), we can observe the autonomic neurovascular bundle of clitoris (***), G- glans, U- urethra, V- vagina, R- rectum.

From this plexus, the autonomic fibers travel along the vaginal wall and the urethra to reach the erectile bodies of the clitoris. The specific autonomic nerves destined for the clitoris are the cavernous nerves (Figure-15). These are small, delicate fibers that travel along the posterolateral aspect of the urethra and the vaginal wall. They penetrate the tunica albuginea of the *corpora cavernosa* and the vestibular bulbs. They regulate the smooth muscle tone of the deep clitoral arteries and the trabecular tissue within the erectile chambers.

An interesting recent study provides a quantitative mapping of clitoral innervation and structure (44) The authors shows that while the absolute number of nerve fibers is ~60% lower in the clitoris, albeit with a higher proportion of myelinated fibers, the innervation density is markedly higher than in the penis. These data will inform not only our understanding of differences and similarities between female and male genitals and their sensory function but also reveal some of the neglected aspects of female sexuality. The original figures in this paper are very interesting and shows the nerves distribution around the clitoris, specially in the glans (44).

In 2026 some important papers show anatomical aspects about the clitoris innervation (19). Previous textbooks suggested the dorsal nerve of the clitoris (DNC) simply tapered off as it reached the glans. The 2026 mapping reveals that the DNC actually branches out into a complex, tree-like structure within the glans, maintaining significant diameter (0.2 mm to 0.7 mm) even at distal points (19). New data shows that clitoral nerves are not confined strictly to the glans. Sensory branches extend significantly into the clitoral hood and the mons pubis. This suggests that surgeons must rethink "safe zones" in aesthetic procedures like hoodectomies (19, 25). A study by Zdilla (25) identified a previously undescribed anatomical subdivision called the "clitoral collar"—a distinct band of preputial tissue around the neck of the clitoris that likely plays a role in stabilizing the neurovascular bundle. Research published by Wolf-Vollenbröker (28) found that clitoral innervation is often not bilaterally symmetrical, which is a critical consideration for reconstructive microsurgery.

Female genital cosmetic surgery has gained significant importance in both plastic surgery and gynecology, with labia minora reduction and clitoral hood reduction among the most common procedures. Recent literature emphasizes the need for precise surgical techniques involving the clitoris, given its dual role in aesthetics and sexual function with careful manage of the "safe zones" of the clitoral complex (40, 45). In clitoral hoodectomy the excision should be limited to the redundant skin of the prepuce. Dissection must remain subcutaneous, avoiding the deep dorsal fascia where the neurovascular bundle resides. In labiaplasty when extending the reduction toward the superior commissure, the surgeon must be wary of the "nerve entry point" near the clitoral body (30). The dorsal midline of the clitoral body is considered a high-risk area. Most modern techniques advocate for lateral incisions or "Y-V" plasty to avoid the central neurovascular highway (28, 30).

## ROLE OF THE CLITORIS IN FEMALE ORGASM

Clitoris is the only human organ evolved for the sole purpose of providing sexual pleasure. While historically overshadowed by vaginal-centric theories of reproduction, modern anatomical studies—particularly since the late 1990s—have redefined it as the powerhouse of the female orgasm (16, 19). The most significant shift in understanding the clitoris is the realization that what is visible externally (the glans) is merely the tip of the iceberg.

The Glans contains approximately 8,000 to 10,000 sensory nerve endings, a density higher than any other part of the human body, including the penis (29, 32, 43). The internal complex beneath the surface, the clitoris extends into two crura (legs) and two vestibular bulbs. These structures hug the vaginal wall and engorge with blood during arousal. Because the internal parts of the clitoris wrap around the vagina, most "vaginal" orgasms are actually the result of indirect stimulation of the clitoral internal structures.

The clitoral orgasm is a complex neuro-vascular event. It follows a specific physiological sequence: Vasocongestion: Upon stimulation, the dorsal and deep arteries increase blood flow to the corpora cavernosa and vestibular bulbs. This causes the clitoris to double or triple in volume. The Reflex Arc: Sensory input travels via the pudendal nerve to the sacral spinal cord and then to the brain's pleasure centers (such as the paraventricular nucleus). The Climax: At the peak of arousal, a series of rhythmic contractions (every 0.8 seconds) occur in the pelvic floor muscles (ischiocavernosus and bulbospongiosus), releasing the accumulated neuromuscular tension (16, 19). We can observe in Figure-16 the physiological sequence of the female orgasm and the role of clitoris.

**Figure 16:**
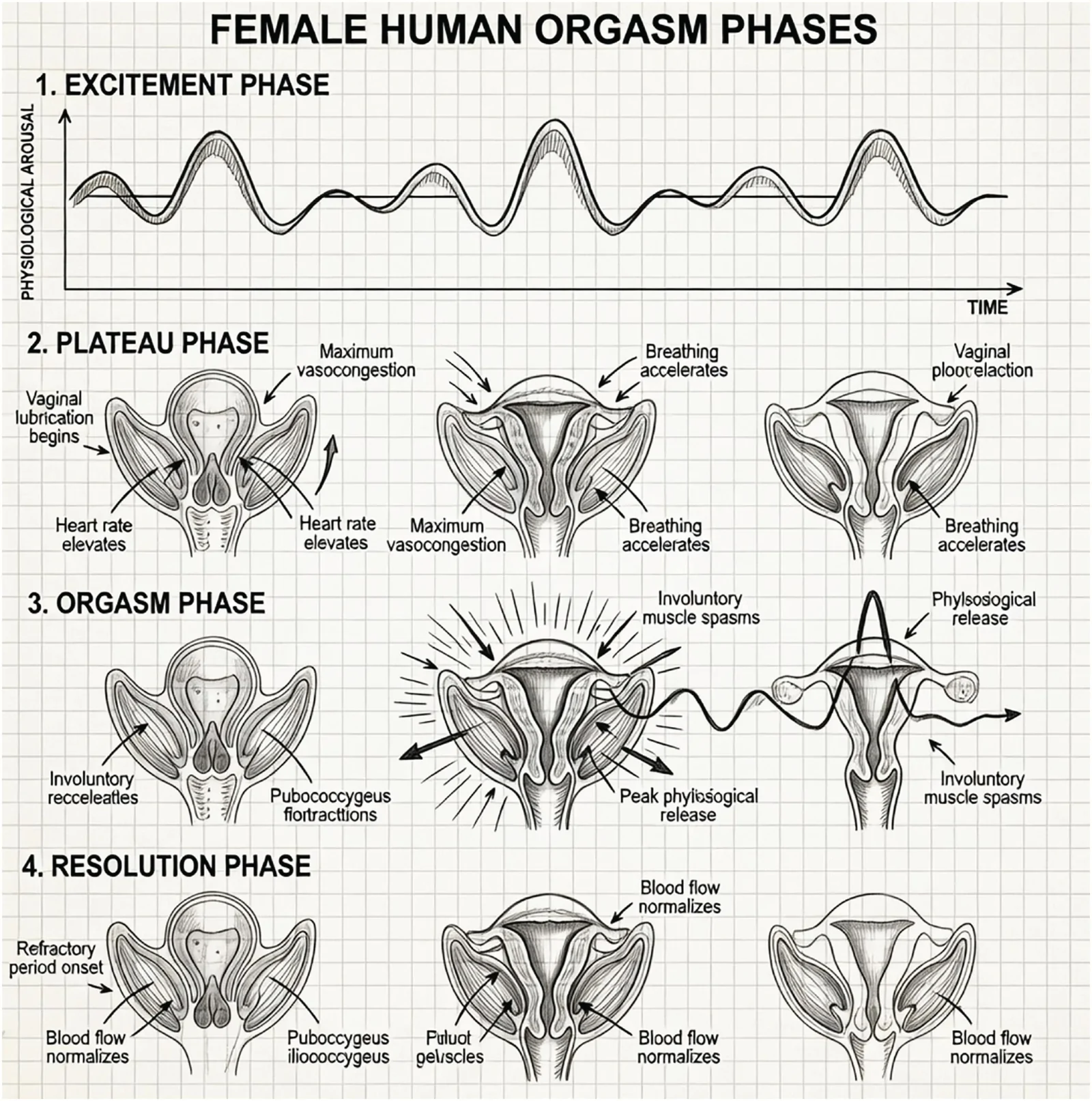
Clitoris and Orgasm.

Schematic drawing showing the orgasm phases. PHASE 1 — Excitement (Blood flow ↑↑, Sensitivity ↑). In this phase we observe a high neural activation with glans erection, clitoris body engorgement, initial expansion of vagina and lubrification; PHASE 2 — Plateau (Sustained high arousal). In this phase we can observe an extremely clitoris sensibility and glans retraction under hood, perineal muscle tension and faster breathing; PHASE 3 – Orgasm. In this phase we observe a dopamine peak, oxytocin release and intense brain activation with rhythmic contractions of pelvic floor, typically in 3 to 12 pulses, the clitoris (orgasm center) has high nerve density, integrative stimulation and primary pleasure source and PHASE 4 – Resolution. The pelvic muscles present relaxation, we observe arousal decrease, the clitoris return to baseline and may remain sensitive during variable period of time in this phase.

The somatic innervation is the afferent (incoming) limb of the orgasmic reflex (16, 19). Afferent Limb: Sensory signals travel from the glans via the DNC to the Sacral Spinal Cord (S2–S4). Integration: The signal is processed in the spinal cord and sent to the brain (sensory cortex). Efferent Limb: If the threshold is reached, the spinal cord sends a motor signal back via the perineal branch of the pudendal nerve to the pelvic floor muscles (ischiocavernosus and bulbospongiosus), causing the rhythmic contractions characteristic of climax.

## CLITORIS AND AESTHETIC SURGERY

Clitoris anatomy knowledge is a prerequisite for understanding his function. These findings are being applied immediately in three areas (46, 47): Providing a roadmap to restore sensation for survivors of female genital mutilation; Gender-Affirming Surgery: Improving the "gold standard" for clitoroplasty by ensuring the neurovascular bundle is mobilized without damaging the newly mapped "tree" branches and Pelvic Cancer Radiotherapy: Using the 3D maps to spare clitoral nerves from radiation damage, preserving sexual function after cancer treatment.

### 1. Clitoral Hood Reduction (Hoodectomy)

This is the most common aesthetic procedure (30, 45). It addresses "clitoral unmasking" or redundant preputial skin that can cause discomfort or hygiene issues. Extended Lateral Wedge Technique: Instead of a simple vertical excision (which carries a risk of midline scarring over the glans), a wedge is removed from the lateral aspects of the hood. This preserves the central neurovascular highway. Y-V Plasty: Used when the hood is narrow but long. A Y-shaped incision is converted into a V-shape to shorten the hood while maintaining the protective function of the skin over the glans. Safety Landmark: Surgeons must remain superficial to the Buck's fascia, where the dorsal nerves are located.

### 2. Nerve-Sparing Clitoroplasty (For Clitoromegaly)

Used in cases of clitoral enlargement (hypertrophy), the goal is to reduce size without sacrificing sensitivity. Reduction and Recession: Rather than removing tissue, the clitoral body is "folded" (plicated) or shortened by resecting a middle segment of the *corpora cavernosa*. Dorsal Nerve Mobilization: The most critical step. The surgeon carefully dissects the neurovascular bundle away from the erectile tissue before any reduction is performed. Glans Preservation: The glans is reduced only if absolutely necessary, usually through a small ventral wedge resection that avoids the high-density dorsal nerve "tree".

### 3. The Foldes Technique (Reconstruction after FGM/C)

This is the gold standard for reconstruction in survivors of Female Genital Mutilation/Cutting (30, 33, 34). Uncovering the Stump: The surgeon identifies the buried clitoral body (the "stump") behind the scar tissue. Ligamentous Release: The suspensory ligament is partially cut to allow the buried portion of the clitoris to be brought forward. Transposition: The healthy, sensitive tissue is moved to the surface to create a new visible glans. This technique restores erogenous sensation in the majority of patients by reconnecting the internal anatomy with external stimulation.

### 4. V-Plasty for Labial-Clitoral Integration

Often performed during labiaplasty, this technique ensures the labia minora and the clitoral hood transition smoothly. Superior Commissure Refinement: By creating a V-shaped flap where the labia meet the hood, surgeons avoid the "dog-ear" deformity (bunched skin) and protect the frenulum of the clitoris. Functional Importance: Maintaining the frenulum is essential for the "tugging" sensation that transmits labial movement to the clitoral glans during sexual activity.

## CLITORIS AND TRANSGENDER SURGERY

The preservation or construction of the clitoris (clitoroplasty) is often considered the "gold standard" for success in gender-affirming surgeries, particularly in feminizing genitoplasty (vaginoplasty) (48-50).

In feminizing surgeries, the clitoris is not merely an aesthetic addition but the primary organ for sexual gratification and sensory feedback. The transition from a phallus to a vulva requires a sophisticated "repurposing" of erectile tissue. Sensation Preservation: The dorsal nerve of the penis is homologous to the dorsal nerve of the clitoris. Successful surgery relies on the mobilization of the neurovascular bundle to ensure the neo-clitoris retains its sensitivity. Psychological Well-being: Research indicates that the presence of a functional, aesthetically pleasing clitoris significantly correlates with reduced gender dysphoria and improved post-operative quality of life.

The most common technique for creating a clitoris in trans women is the glans-based clitoroplasty. The Neurovascular Bundle (NVB) is the lifeline of the neo-clitoris. During surgery, the glans penis is reduced in size while remaining attached to its original nerve and blood supply (the dorsal arteries and nerves). This pedicle is then tunneled and positioned superior to the neo-urethral orifice. A critical challenge is the partial resection of the *corpora cavernosa*. While some erectile tissue is removed to create a feminine contour, enough must be preserved to allow for the physiological engorgement of the neo-clitoris during arousal, mimicking cisgender female anatomy.

The importance of the clitoris is further highlighted by the risks associated with its surgical construction: Glans Necrosis: If the blood supply via the dorsal arteries is compromised during the stripping of the penile shaft, the neo-clitoris may undergo necrosis. Desensitization: Excessive tension on the neurovascular bundle or direct trauma to the dorsal nerves can lead to permanent loss of erogenous sensation. Chronic Pain: Improper burial of the nerves can lead to the formation of neuromas or chronic hypersensitivity.

### Metoidioplasty

In transmasculine individuals, the clitoris itself is the primary focus of metoidioplasty. Through testosterone therapy, clitoral hypertrophy (clitoromegaly) occurs. Surgery involves releasing the clitoral ligaments (suspensory and chordee) to allow the enlarged clitoris to project forward, functioning as a small phallus. This procedure preserves the high density of sensory nerve endings, ensuring that the patient maintains peak sexual sensation (51, 52).

In the context of gender-affirming surgery for transmasculine individuals, metoidioplasty is a surgical option that relies entirely on the pre-existing anatomical structures of the clitoris, which have been modified by androgen therapy (testosterone). Unlike phalloplasty, which uses tissue grafts, metoidioplasty focuses on the "release" and repositioning of local tissues (51, 52).

The foundation of a metoidioplasty is the androgen-induced clitoromegaly. Before surgery, patients typically undergo 1–2 years of testosterone therapy, which causes the clitoral glans and *corpora cavernosa* to hypertrophy. The final length of the "metoidio-phallus" is strictly limited by the individual's biological response to testosterone. These erectile bodies remain functional, allowing the neo-phallus to become engorged during arousal, preserving the patient's natural erectile capacity. A key anatomical barrier to the projection of the clitoris is the suspensory ligament and the ventral tethering known as chordee.

The most significant anatomical priority in metoidioplasty is the protection of the dorsal neurovascular bundle. Because the nerves are located on the dorsal aspect (top) of the clitoral body, surgeons must be extremely precise during the ligamentous release. Preservation of the deep and dorsal arteries ensures that the hypertrophied tissue remains viable and responsive to stimulation. The high success rate of erogenous sensation post-metoidioplasty is due to the minimal manipulation of these specific nerves compared to other phallic constructions.

In cases of clitoromegaly—the abnormal enlargement of the clitoris—surgical intervention via clitoroplasty is often indicated when the condition causes physical discomfort, psychological distress, or functional interference. Modern surgical philosophy has shifted from "clitoral reduction" to "clitoral preservation," prioritizing the maintenance of neurovascular integrity.

Clitoromegaly can result from various conditions, and the surgical approach must consider the underlying cause: Congenital Adrenal Hyperplasia (CAH): The most common cause, where excess androgens lead to virilization of the genitalia (Figure-17); Polycystic Ovary Syndrome (PCOS): Severe cases can lead to mild-to-moderate enlargement.; Androgen-secreting tumors: Rapid onset of enlargement often points to adrenal or ovarian tumors and Exogenous Steroids: Use of anabolic steroids can lead to irreversible clitoral growth (51, 52).

**Figure 17:**
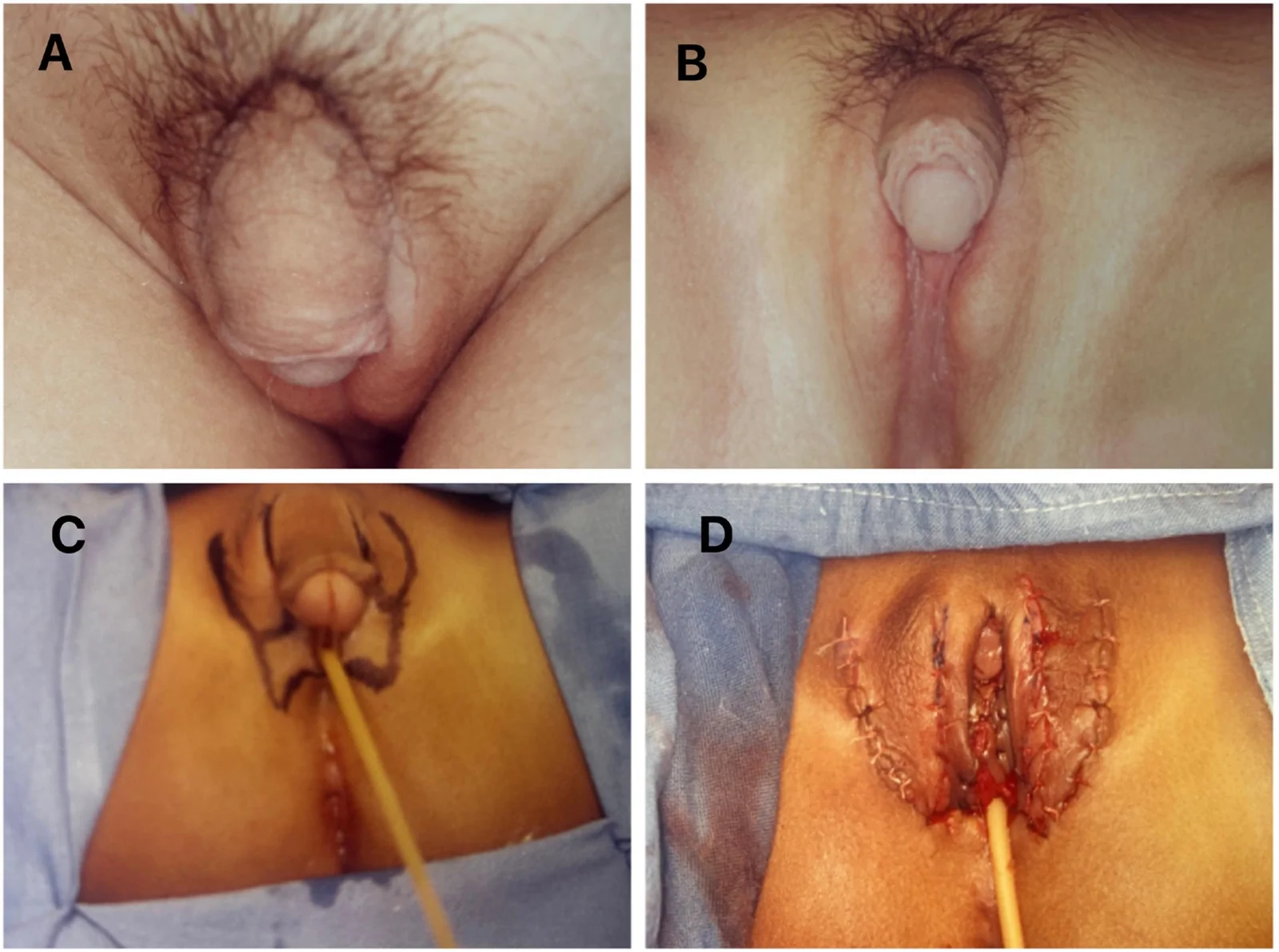
Clitoris and Orgasm.

A) Clitoromegaly in patient with 1 year-old and CAH; B) A view of the perineal region of the same patient; C) Other patient 9 months-old with CAH during the preparation for the surgical correction and D) Final aspect of this patient after citoroplasty.

Historically, clitoromegaly was often treated with a clitorectomy (total removal), which resulted in permanent loss of sexual sensation. Contemporary techniques focus on reduction and recession while preserving the glans and its innervation. Degloving: An incision is made around the clitoral hood and the skin is "degloved" to expose the clitoral body and the neurovascular bundles (NVB). Mobilization of the NVB: Utilizing the anatomical data confirmed in recent 2026 studies, the surgeon carefully separates the dorsal nerves and arteries from the erectile bodies (*corpora cavernosa*). Partial Corporoplasty: A portion of the erectile tissue is excised or plicated (folded) to reduce the length and girth of the clitoral shaft. Glans Reduction: If the glans itself is excessively large, a "wedge resection" may be performed. However, surgeons must be extremely conservative here to avoid damaging the high-density nerve endings mapped in the glans. Recession and Fixation: The shortened clitoris is recessed back toward the pubic symphysis to create a more feminine and comfortable anatomical position.

The success of a clitoroplasty is measured by three main pillars: Sensation: Post-operative testing should ideally show preserved erogenous sensitivity. Aesthetics: The goal is a clitoris that is proportional to the labia majora and minora, typically partially covered by the clitoral hood. Function: Reducing the size of the clitoris can alleviate "chafing" or pain caused by friction against clothing, which is a common complaint in patients with significant clitoromegaly.

## CONCLUSION

The clitoris is a very important structure to female physiology, specially the orgasm. The knowledge of clitoris anatomy is the fundamental step in aesthetic surgery and in reconstruction, specially in transgender procedures.

## Data Availability

All data generated or analysed during this study are included in this published article
